# Loss of lysosomal acid lipase contributes to Alzheimer's disease pathology and cognitive decline

**DOI:** 10.1002/alz.70486

**Published:** 2025-07-18

**Authors:** Alexandra M. Barnett, Elizabeth M. McNair, Lamar Dawkins, Jian Zou, Viktoriya D. Nikolova, Sheryl S. Moy, Greg T. Sutherland, Julia Stevens, Meagan Colie, Kemi Katemboh, Hope Kellner, Katherine Ho, Corina Damian, Sagan DeCastro, Ryan P. Vetreno, Leon G. Coleman

**Affiliations:** ^1^ Department of Pharmacology University of North Carolina at Chapel Hill School of Medicine Chapel Hill North Carolina USA; ^2^ Bowles Center for Alcohol Studies University of North Carolina at Chapel Hill School of Medicine Chapel Hill North Carolina USA; ^3^ Department of Psychiatry University of North Carolina at Chapel Hill School of Medicine Chapel Hill North Carolina USA; ^4^ Carolina Institute for Developmental Disabilities University of North Carolina at Chapel Hill Carrboro North Carolina USA; ^5^ New South Wales Brain Tissue Resource Centre and Charles Perkins Centre School of Medical Sciences Faculty of Medicine and Health The University of Sydney Camperdown New South Wales Australia

**Keywords:** aging, alcohol, Alzheimer's disease, lipid, lysosomal acid lipase, lysosome, obesity

## Abstract

**INTRODUCTION:**

Underlying drivers of Alzheimer's disease (AD) remain unknown, though several distinct risk exposures share a common pathological progression.

**METHODS:**

The cellular and molecular consequences of two common midlife AD risk factors—heavy alcohol use and obesity—were compared to uncover novel mediators that contribute to AD.

**RESULTS:**

Both AD risk exposures reduced levels of neuronal lysosomal acid lipase (LAL), which contributed to AD pathology and cognitive decline. LAL was lost with age in mice and humans with greater losses in AD and inverse associations with amyloid β (Aβ). LAL loss preceded Aβ pathology in AD mice, and neuronal LAL knockdown enhanced pathology and cognitive decline. In human AD brain, robust reductions in LAL protein were found with indications of a transcriptional mechanism. LAL gene therapy reduced pathology and improved cognition and affect in vivo.

**DISCUSSION:**

LAL loss is an age‐related contributor to AD pathology that can be targeted therapeutically.

**Highlights:**

- The loss of lysosomal acid lipase (LAL) contributes to Alzheimer's disease progression.
- LAL is lost is normal aging and Alzheimer's disease risk exposures.
- LAL loss is greater in human Alzheimer's brain and predicts the extent of pathology.
- LAL gene therapy blunts Alzheimer’ pathology, improving cognition and mood with age.

## 1 BACKGROUND

Alzheimer's disease (AD) is the most common form of dementia with over 90% of cases being sporadic or late‐onset AD (LOAD). Though the etiology of LOAD is often unknown, risk factors such as heavy smoking or alcohol use, diabetes, hypertension, and obesity account for much of the risk.^1^, ^2^, ^3^, ^4^ Though these exposures have diverse biological impacts, they converge upon a shared pathological progression—the accumulation of intraneuronal amyloid β (Aβ), extracellular Aβ plaque deposition, and pathological tau species formation with aging.^5^ Therefore, identifying shared consequences of these AD risk exposures can be used to reveal underlying cellular drivers of LOAD. Several cellular functions have recently been implicated in LOAD such as neuroinflammation, dysfunction of the endosomal and autophago‐lysosomal systems,^6^, ^7^, ^8^, ^9^, ^10^, ^11^, ^12^, ^13^ and altered lipid metabolism.^14^, ^15^, ^16^, ^17^ However, how dysfunction in these systems integrate with each other to result in emergence of LOAD in diverse etiological settings remains unknown.

Intraneuronal Aβ is a feature of LOAD that is associated with early cognitive deficits.^18^, ^19^, ^20^, ^21^, ^22^, ^23^, ^24^, ^25^, ^26^ Aβ normally transits to neuronal endosomes with lysosomal degradation after internalization from the cell surface or endocytosis.^27^, ^28^, ^29^, ^30^, ^31^, ^32^, ^33^ Dysfunction of autophagy or lysosomes has been suggested to promote intraneuronal Aβ accumulation and subsequent plaque formation.^6^, ^7^, ^8^, ^9^, ^10^, ^11^, ^12^ However, the molecular drivers of lysosomal dysfunction associated with LOAD remain unknown. Human studies also suggest altered lipid metabolism. For instance, the polymorphisms in lipid efflux proteins increase risk,^16^, ^17^ and the ε4 apolipoprotein polymorphism is a strong genetic LOAD risk factor.^14^ Lipid metabolism is governed by coordinated actions of lipogenesis, lipolysis, and lysosomal digestion (i.e., lipophagy).^34^, ^35^ However, mechanisms by which aberrant lipid metabolism promote LOAD are unknown.

**RESEARCH IN CONTEXT**

1. **Systematic review**: Underlying drivers of Alzheimer's disease (AD) are unknown, though several risk exposures promote disease. AD risk exposures are diverse; however, they share a common pathological progression.^1^, ^2^, ^3^, ^4^, ^5^ Studies suggest roles for altered fat metabolism and lysosome dysfunction, though therapeutic targets for these processes have not emerged.^6^, ^7^, ^8^, ^9^, ^10^, ^11^, ^12^, ^13^, ^14^, ^15^, ^16^, ^17^
2. **Interpretation**: By comparing two common midlife risk exposures for AD, we found that fat accumulation within neuronal lysosomes contributes to AD pathology. This involved the loss of lysosomal acid lipase (LAL). The accumulation in fat within lysosomes disrupted their function, promoting AD pathology and cognitive decline. In human brain, LAL was lost with age with further decline in AD. LAL neuronal gene therapy blunted AD pathology, improving cognition and mood.
3. **Future directions**: Together, this implicates neuronal LAL loss as an age‐related AD risk factor, and identifies LAL as a promising diagnostic, preventative, and therapeutic target for AD.

To identify underlying drivers of LOAD pathogenesis, we compared the cellular and molecular consequences of two distinct midlife risk factors for LOAD, heavy alcohol use and obesity.^4^, ^36^ A recent nationwide retrospective study found that heavy alcohol use was the strongest modifiable risk factor for dementia and AD (HR ∼ 3 and 2, respectively).^4^ Recent preclinical studies from our group and others are consistent with this epidemiology, finding that chronic alcohol increases Aβ and tau pathology and behavioral deficits.^37^, ^38^, ^39^, ^40^ We recently reported that early life binge alcohol increased intraneuronal AD pathology in adulthood.^38^ This involved proinflammatory microglial activation, though the mechanism underlying neuronal amyloid accumulation remained unknown. Obesity during midlife is also a major risk factor for LOAD, and Western diet in rodents increases hippocampal Aβ plaques pathology and promotes cognitive deficits.^41^, ^42^ Using these two risk exposures to identify shared cellular deficits that underly LOAD pathogenesis, we found that the accumulation of neuronal lysosomal lipid (NLL) contributes to LOAD pathogenesis. This involves the loss of lysosomal acid lipase (LAL) the main lysosomal lipase. A role for LAL extended beyond these risk factors with neuronal LAL loss in normal aging that was greatly enhanced in human LOAD subjects without heavy alcohol use or obesity. Signs of a transcriptional mechanism were found, with altered localization of RNA polymerase II across the LAL gene body in female human LOAD hippocampus. LAL neuronal gene therapy (GT) blunted the enhancement Aβ pathology and cognitive deficits caused by midlife alcohol and prevented cognitive decline and affective dysfunction with aging in AD mice. Thus, LAL loss with aging contributes to the emergence of Aβ that can be targeted therapeutically for the prevention or treatment of AD.

## 2 METHODS

### 2.1 *Post mortem* human brain samples

Brains from human donors were from the New South Wales Brain Tissue Resource Centre (NSW‐BTRC) and the Victoria Brain Bank (VBB) in Australia. Human samples were obtained under Ethics Committee Approval Number X11‐0107^43^, ^44^ in accordance with the ethical standards in the 1964 Declaration of Helsinki. Diversity, equity, and inclusion: No subjects were excluded based on sex, ethnicity, or race. Both males and females were included in this study. Subject demographics are summarized in Table S1.

### 2.2 Mouse models

AD mice were obtained from the Jackson Laboratory (JAX) Mutant Mouse Resource & Research Centers (MMRRC, Supplemental Table 2). 3xTg‐AD (human APPSwe, tauP301, and Psen1^tm1Mpm^, MMRRC stock #034830/JAX #004807)^21^ and 5xFAD (human APPSwFlLon, PSEN1^m146L,L286V^, MMRRC stock #034840/JAX #006554)^45^ were used. Aged C57BL6 wild‐type (WT) mice 9–20 months old were obtained from the Naational Institute on Aging (NIA) aged mouse colony (supplied by Jackson). We did not have approval to receive 3‐month‐old mice through the NIA aged mouse colony. Therefore, 3‐month‐old WT female mice were ordered from Jackson Labs, while males, which were available in our vivarium at the time, were bred in‐house. Mice were bred and pups weaned at 30 days of age and group‐housed with same‐sex littermates. Animal protocols were approved by the University of North Carolina at Chapel Hill Institutional Animal Care and Use Committee (IACUC) and were in accordance with NIH regulations (Protocols 20‐232.0 and 21‐052.0).

### 2.3 Midlife binge alcohol treatment

**FIGURE 1:**
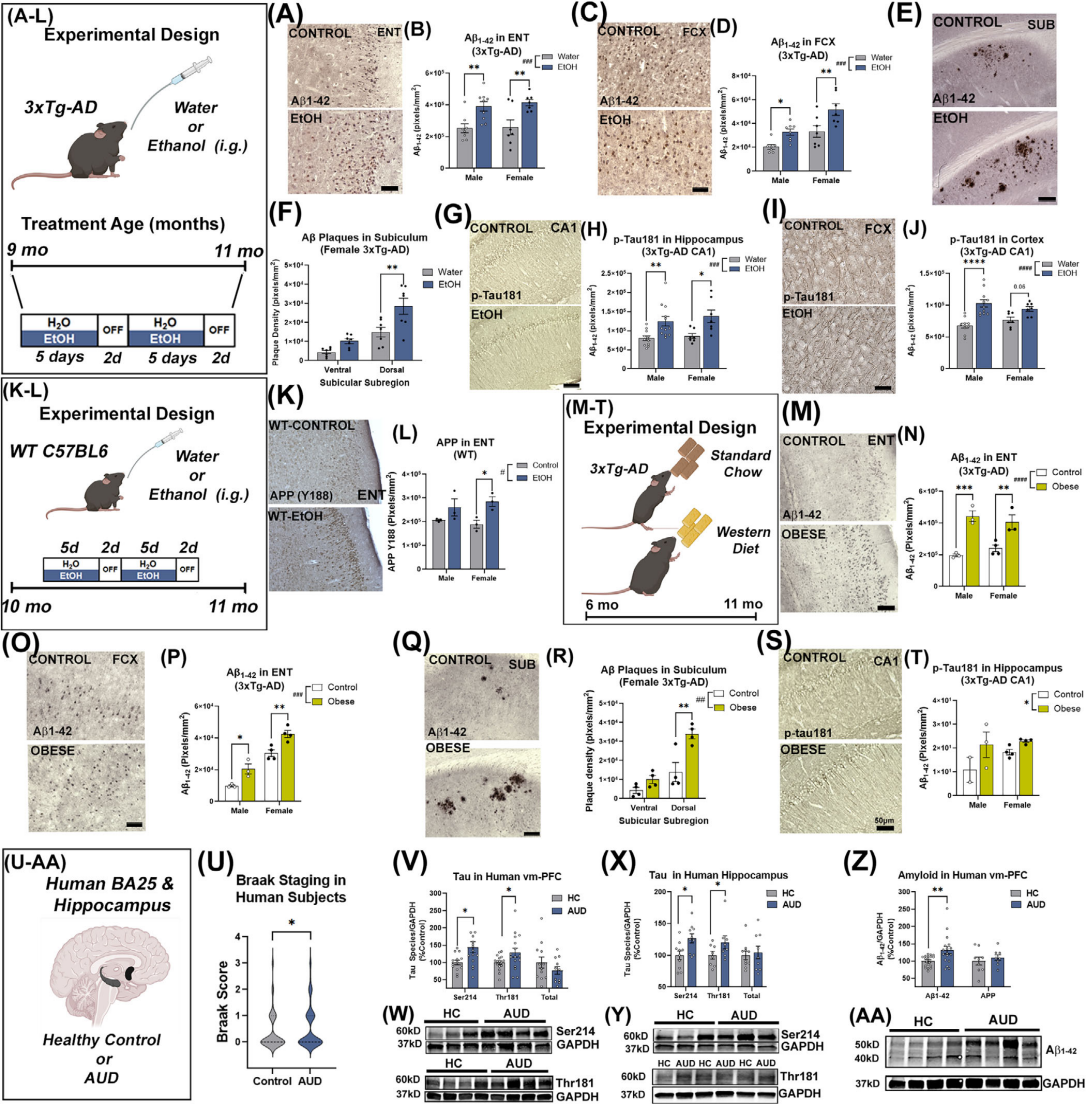
Midlife alcohol and obesity increase LOAD pathology. (A, B) Ethanol increased Aβ1‐42 in ENT (F_1,27 _= 20.4, *###p *= 0.0001) and (C, D) FCX in both sexes (F_1,25 _= 16.7, *###p *< 0.0001), *p < 0.05, **p < 0.01 Sidak's post‐test. *N* = 15 control, 16 ethanol. Scale: 100 µm (E, F) Ethanol increased Aβ1‐42+ plaques in the subiculum of female mice by 91% (dorsal) and 127% (ventral). Scale bar: 200 µm. Ethanol increased p‐tau181 in both sexes in the (G, H) CA1 F_1,33 _= 17.5, ###*p *= 0.0002, and (I, J) FCX F_1,31 _= 27.7, ####*p *< 0.0001. *N* = 18 control, 19 ethanol. Scale bar: 50 µm. (K, L) 5 weeks of ethanol increased total APP in ENT of WT 10‐mo mice. F_1,8 _= 11.1, #*p *= 0.01, *p < 0.05 Sidak's post‐test. (M, N) Midlife obesity increased ENT Aβ1‐42 by 47%. F_1,9 _= 48.2, ####*p *< 0.0001. (O, P) Obesity increased Aβ1‐42 in FCX two‐fold in males and 40% in females. Sex: F_1,10 _= 89.2, *p *< 0.0001, Obesity: F_1,10 _= 25.2, *###p *= 0.0005, *N* = 7/treatment group. (Q, R) 2.4‐fold increase in Aβ plaques in the dorsal subiculum of obese females. Scale bar: 200 µm. *p < 0.05, **p < 0.01, ***p < 0.001, Sidak's multiple comparison test. (S, T) Obese mice had increased p‐tau181 in CA1. F_1,19 _= 6.3, *#p *= 0.03. *N* = 6 Control, 7 obese. One control male hippocampus excluded due to damage by hydrocephalus. (U) Braak staging scores were slightly increased in AUD compared to age‐match moderate‐drinking controls. *p < 0.05, paired *t‐*test. (V, W) Human AUD vm‐PFC had a ∼50% increase in p‐tau214 and a ∼30% increase in p‐tau181 with no change in total tau versus age‐matched controls. **p < 0.01, paired *t‐*test, *N* = 16/group. (X, Y) Human AUD hippocampus showed a 27% increase in p‐tau214 and a 20% increase in p‐tau181 by Western blot. **p < 0.01. (Z, AA) Aβ1‐42 was increased in AUD vm‐PFC by 31%. *N* = 17/group. *t*‐test. *p < 0.5 **p < 0.01 ***p < 0.001. Males, open circles; females, closed circles.

**FIGURE 2:**
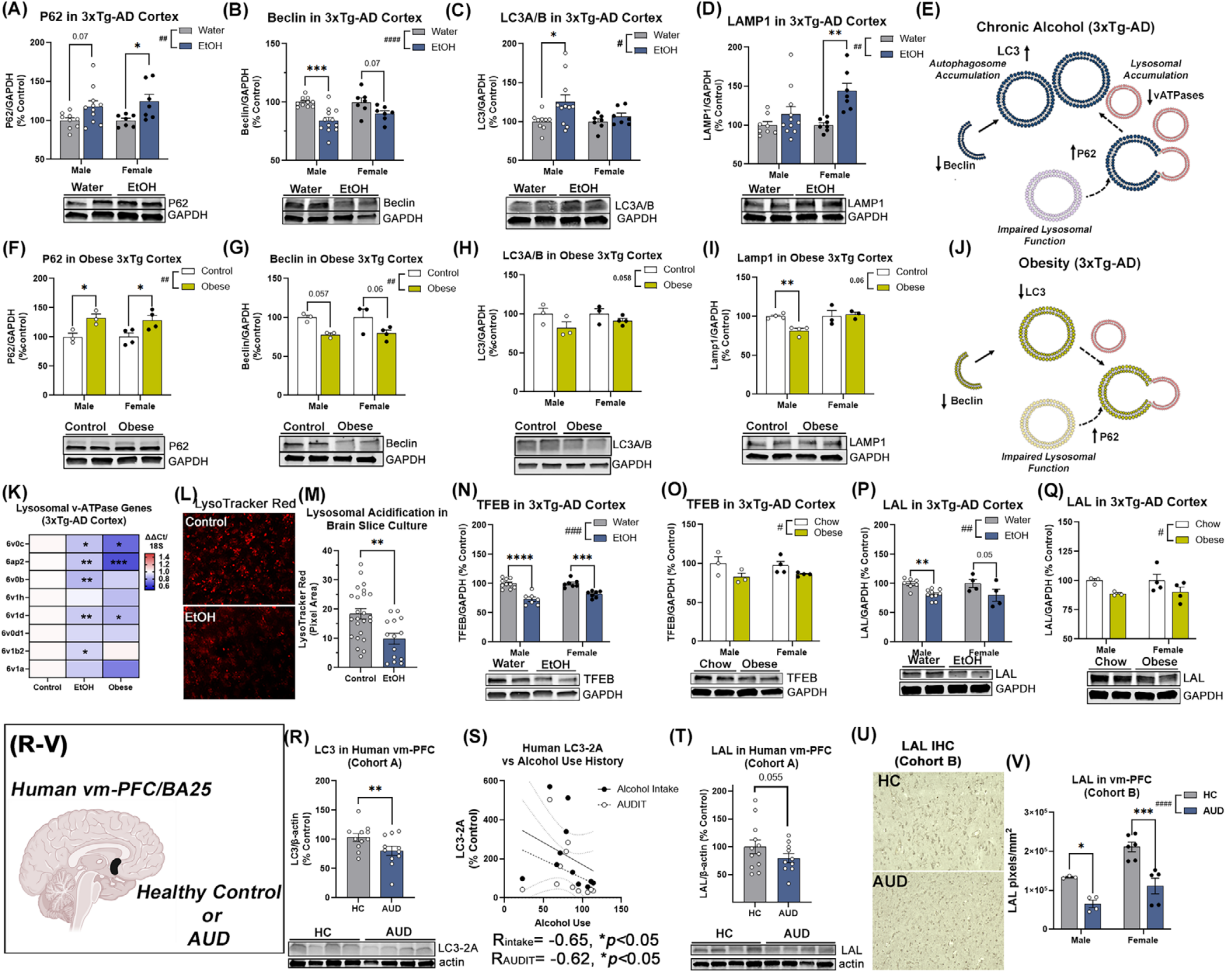
Ethanol and obesity disrupt autophagic flux and lysosomal function in 3xTg‐AD mice. Ethanol (A) increased P62 (F_1,30 _= 11.2, ##*p *= 0.002), (B) reduced Beclin‐1 (F_1,32 _= 21.1, ####*p *< 0.0001) (C) increased LC3 (F_1,30 _= 5.2, #*p *= 0.03), and (D) increased LAMP1 (F_1,29 _= 12.6, ##*p *= 0.001) in 3xTg‐AD cortex. *N* = 15 control, 18 ethanol. *p < 0.05, **p < 0.01, ****p < 0.0001, Sidak's post‐tests. Males, open circles; females, closed circles. (E) Findings from A‐D support lysosomal dysfunction. Obesity (F) increased P62 (F_1,10 _= 17.2, ##*p *= 0.002), and (G) reduced Beclin (F_1,9 _= 13.3, ##*p *= 0.005), with (H) a trend toward reduced LC3 (F_1,9 _= 11.2, *p *= 0.06) in 3xTg‐AD cortex. (I) No change in LAMP1 in obese 3xTg‐AD cortex. *N* = 6 control, 7 diet‐induced obesity. *p < 0.05, **p < 0.01, ***p < 0.001, Sidak's post‐test. (J) Findings from (F‐I) support lysosomal dysfunction. (K) Reduced expression of lysosome acidifying v‐ATPases genes by both ethanol and obesity. (L, M) Lysosomal acidification in HEBSC slice cultures was reduced by ethanol (100 mM, 4 days) by 50% as measured by LysoTracker red. TFEB protein was reduced by (N) ethanol (F_1,27 _= 62.5, ####*p *< 0.0001) and (O) obesity (F_1,10 _= 7.2 #*p *= 0.02) in 3xTg‐AD cortex. (P) Reductions in LAL protein caused by ethanol (F_1,21 _= 14.2, ##*p *= 0.001) and (Q) obesity (F_1,10 _= 6.4 #*p *= 0.03). **p < 0.01, ***p < 0.001, ****p < 0.0001, Sidak's post‐tests (R, V) Altered autophagy and reduced LAL in post‐human AUD vm‐PFC/BA25 in two cohorts. (R) LC3‐II was reduced by ∼20% in AUD vm‐PFC and (S) was correlated negatively with lifetime alcohol use and the Alcohol Use Disorders Identification Test (AUDIT) score. (T) In human cohort A, reduced LAL was found in AUD by Western blot that approached the statistical significance threshold. *p *= 0.055. (U, V) IHC on subjects from cohort B found ∼50% reductions in LAL in male and female human AUD vm‐PFC. F_1,14 _= 30.2, ####*p <* 0.0001. *p < 0.05, ***p < 0.001, Sidak's post‐tests.

**FIGURE 3:**
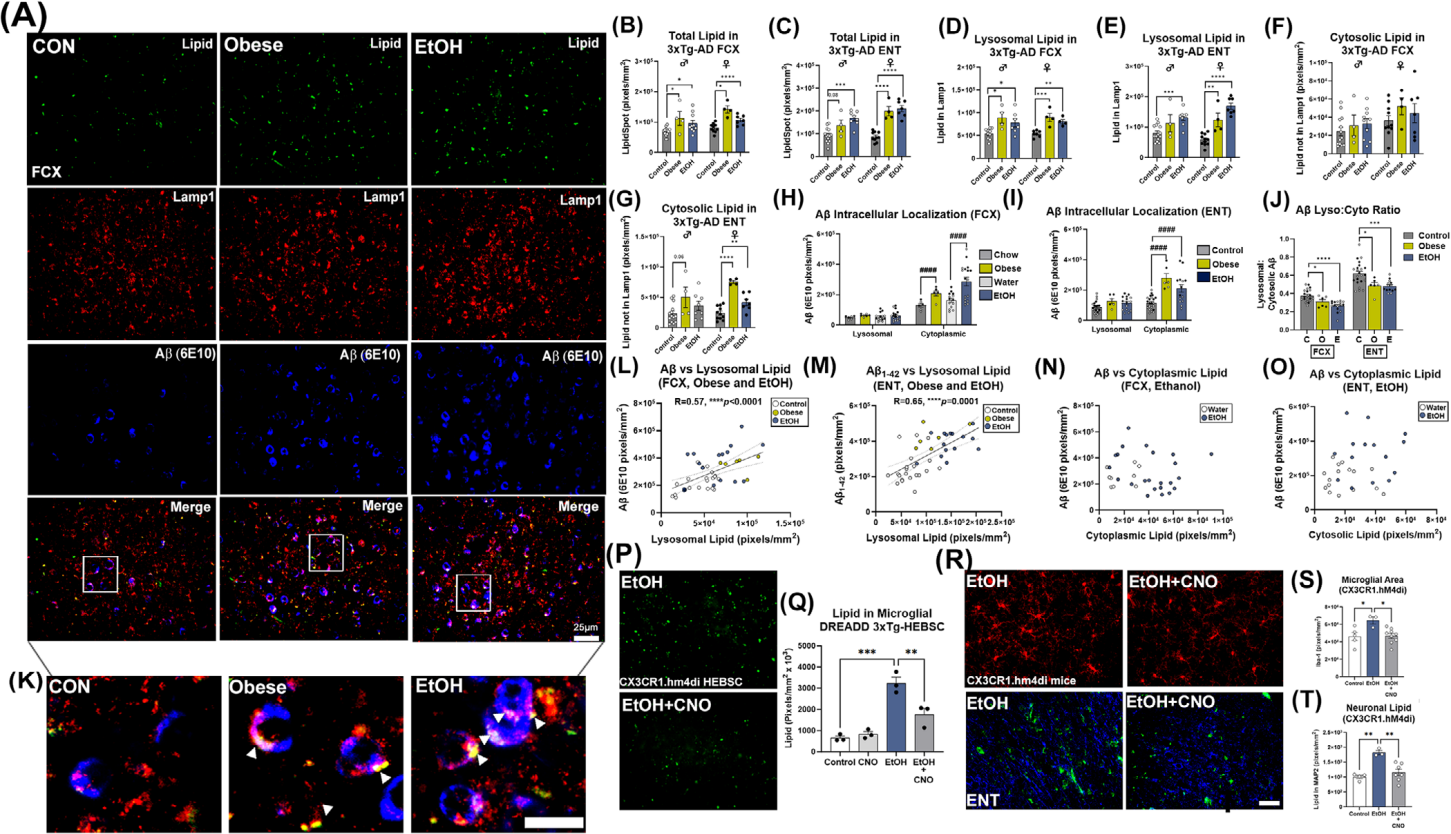
Increased lysosomal lipid with obesity and ethanol that is enhanced by proinflammatory microglia. (A) Triple immunofluorescence (IF) for cytosolic lipid (LipidSpot, green), LAMP1 (red), and Aβ (blue). Normal chow and water gavage cohorts were combined since no differences were present. Scale bar: 25 µm. (B) Intracellular lipid was increased in FCX by obesity and ethanol in males (treatment effect F_2,24 _= 5.2, *p *= 0.01) and females (F_2,18 _= 19.1, *p* < 0.0001) as well as in the (C) ENT in males (F_2,23 _= 12.1, *p* = 0.0003) and females (F_2,18 _= 39.4, *p* < 0.0001). (D) Lysosomal lipid was increased in FCX by obesity and ethanol in males (F_2,18 _= 6.2, *p *= 0.009) and females (F_2,12 _= 14.9, *p *= 0.009) and in the (E) ENT of males (F_2,23 _= 6.8, p = 0.005) and females (F_2,19 _= 36.3, *p* < 0.0001). (F) Non‐lysosomal cytosolic lipid was unchanged in the FCX, with (G) increases in the female ENT (F_2,19 _= 30.1, *p* < 0.0001). Increases in total Aβ (6E10) were found in the extra‐lysosomal cytoplasm in (H) FCX by obesity (61%, F_1,20 _= 19.52, *p = *0.0003) and ethanol (1.8‐fold, F_1,59 _= 14.38, *p = *0.0004) as well as in the (I) ENT (obese: 2.2‐fold, ethanol: 1.9‐fold, F_2,82 _= 20.38, *p = *0.00001). (J) Reduction of lysosomal:extra‐lysosomal (lyso:cyto) Aβ ratios. FCX: F_2,41 _= 14.50, *p *< 0.0001, ENT: F_2,40 _= 8.788, *p *= 0.0007. Sexes combined due to lack of sex difference. Males, open circles; females, filled circles. (K) High magnification image showing increased lysosomal lipid within Aβ+ neurons. Scale: 10 µm. (Key: A‐J) Open circles‐males, filled circles‐females. *p < 0.05, **p < 0.01, ***p < 0.001, ****p < 0.0001, Dunnett's post‐test. Strong positive correlations of lysosomal lipid with total Aβ in (L) FCX and (M) Aβ1‐42 in ENT with obesity and ethanol. (N‐O) No correlation was found between cytoplasmic lipid and Aβ in the FCX or ENT. (P, Q) Ethanol‐induced increases in cytosolic lipid in HEBSCs was prevented by microglial inhibition (hM4di). (R) Representative images finding chemogenetic inhibition of proinflammatory microglia (CX3CR1Cre^ERT2^.hM4di‐DIO) prevented ethanol‐induced (S) microglial activation and (T) NLL accumulation in the ENT. *p < 0.05, **p < 0.01, ***p < 0.001, ****p < 0.0001, Sidak's post‐test.

3xTg‐AD or WT mice, mice received a single daily intragastric (i.g.) administration of either alcohol (i.e., ethanol [EtOH]) or water (5‐days on/2‐days off) to mimic human intermittent drinking patterns during midlife (9 months of age) for 5–8 weeks. The pharmacokinetics of the ethanol paradigm have been deeply characterized in both mice and humans. Mice metabolize ethanol ∼8 x faster than humans do.^46^ Cross species comparisons find this dose results in an EtOH area under the curve (AUC) of 230.7mM x hour, which is equivalent to ∼1.4 g/kg in humans (∼5–7 standard drinks/70 kg person) which is within the binge consumption range.^47^ As a measure for changes in systemic metabolism with chronic ethanol, body weights were tracked across the experiments. No differences in body weight were found between treatment groups. Mice were sacrificed 24 hours after final administration of ethanol or after the last behavioral assessment and tissue was collected for tissue analyses. Two separate cohorts of midlife ethanol treatment were performed for Figures 1, 2, 3.

### 2.4 Western diet‐induced midlife obesity

3xTg‐AD or WT mice were fed either a complete control diet (PicoLab 50 IF/6F, 3.6 kcal/g, 14.8% calories from fat) or a Western diet (Envigo TD.88137, 4.5 kcal/g, 42% calories from fat) to induce obesity (Supplemental Table 2). Given the ∼6‐ to 8‐week delay between initiation of Western diet and a clear obese phenotype, Western diet was started at 6 months of age so mice would be obese during the 9‐ to 11‐month‐old midlife period. Mice were sacrificed and tissue was collected for analysis.

### 2.5 Microglial Gi DREADD inhibition

For ex vivo slice culture experiments HEBSCs from 3xTg‐AD mice were transfected with hM4di into microglia (AAV9.CD68.hM4di for 24 hours) as previously reported^45^ and then treated with ethanol (100 mM, 4 days) ± CNO (1 µM). For in vivo studies, heterozygous CX3CR1.Cre^ERT2^.hM4di were treated with tamoxifen (75 mg/kg/d, i.p.) for 5 days during adulthood (12 weeks of age). After 4 weeks to allow for repopulation of peripheral monocytes, mice received either ethanol (5 g/kg/d, i.g.) or water for 10 days ± CNO (3 mg/kg, i.p., given 10 hours after ethanol) and sacrificed 24 hours after the last ethanol treatment.

### 2.6 Perfusion and tissue collection for immunohistochemistry

At the conclusion of each experiment, subjects were sacrificed by transcardial perfusion with 0.1 M phosphate‐buffered saline (PBS, pH 7.4), and brains excised and hemisected. On one hemisphere, the cortex and hippocampus were dissected and snap frozen in liquid nitrogen for protein and RNA analyses as we have reported previously.^38^ The other hemisphere was drop‐fixed in 4.0% paraformaldehyde for immunohistochemical assessments. Coronal sections were cut (40 µm) on a sliding microtome (MICROM HM450; ThermoScientific, Austin, Texas, USA), and sections were sequentially collected into well plates and stored at ‐20°C in cryoprotectant (30% glycol/30% ethylene glycol in PBS).

### 2.7 Real‐time polymerase chain reaction

The mRNA was extracted from frozen cortex or hippocampus as reported.^38^ Briefly, samples were homogenized with Trizol (Invitrogen) and RNA was isolated by chloroform extraction, followed by reverse transcription as described previously.^48^ SYBR green polymerase chain reaction (PCR) master mix (Applied Biosystems, Foster City, California, USA) was used for quantitative real‐time PCR (qRT‐PCR) analysis. Primer sequences were designed using the National Library of Medicine Primer‐BLAST tool or were obtained from PrimerBank database (Table S2).^49^ Only primers with no predicted non‐specific targets and single peak melt curves were used. Genes of interest were normalized to the expression of the reference gene 18S using the cycle threshold (Ct) value of each target gene product. The ΔΔCt method was used to compare relative differences between control and treatment groups, and the ratio by 18S or the percent change relative to 18S were used in analysis.

### 2.8 Western blot

Western blot was performed as we have reported previously.^38^, ^50^ Brain tissue from the cerebral cortex was homogenized in lysis buffer (Tris‐HCl, pH 7.5, sucrose, ethylenediaminetetraacetic acid [EDTA], ethylene glycol‐bis(β‐aminoethyl ether)‐N,N,N',N'‐tetraacetic acid [EGTA], 1% Triton X‐100, protease, and phosphatase inhibitors). Forty milligrams of protein were loaded into each lane on sodium dodecyl sulfate (SDS) polyacrylamide gels and were transferred to polyvinylidene fluoride (PVDF) membranes. Membranes were washed in Tris‐buffered saline (TBxS) and blocked for 1 hour at room temperature (Li‐Cor Blocking Solution; 92760001) then incubated overnight at 4°Celsius with the primary antibodies listed in Table S2. Membranes were washed in TBS with 0.1% Tween‐20 (Sigma‐Aldrich, St. Louis, Missouri, USA) then were incubated in the appropriate conjugated secondary antibody (Rockland H&L Pre‐absorbed). Membranes were washed again in TBS and visualized using LiCor Image Studio Lite Ver 5.2. Western Blots were analyzed using Image Studio Lite software and each protein of interest was normalized to housekeeping protein glyceraldehyde‐3‐phosphate dehydrogenase (GAPDH). GAPDH expression was not affected by ethanol treatment or high‐fat diet. The protein of interest was normalized to GAPDH for each sample and the percent change relative to controls was calculated for each blot.

### 2.9 Immunohistochemistry and immunofluorescence

Free‐floating sections (40 µm) were washed in 0.1 M PBS, quenched in 0.6% H_2_O_2_ for 30 min to inhibit endogenous peroxidases, then incubated at 70°C in pH = 6.0 1x citrate buffer for antigen retrieval. To allow membrane permeabilization, sections were blocked for 1 hour at room temperature in 4% normal serum with 0.1% Triton X‐100. Sections were incubated in the relevant primary antibody (please see above) in blocking solution at 4°C overnight. Negative controls for non‐specific binding were done using the same protocol, omitting the primary antibody. The next day sections were incubated at room temperature for one hour with a biotinylated secondary antibody (1:200, Vector Laboratories, Burlingame, California, USA), washed briefly in PBS, then incubated for 1 hour in avidin–biotin complex (ABC) (Vectastain ABC Kit; Vector Laboratories). Nickel‐enhanced diaminobenzidine (Sigma‐Aldrich) was used as the chromogen for visualization. Sections were mounted on charged glass slides, allowed to dry, dehydrated in a series of ethanol, and covered using Cytoseal (Fischer). For immunofluorescence (IF), the day after primary antibody incubation, sections were washed with PBS and incubated for 1 hour at room temperature with the respective Alexa Fluor conjugated secondary antibodies (1:1000; Invitrogen, Carlsbad, California, USA). If staining for neutral lipid droplets was being performed, sections were washed with PBS then stained with 1xLipidSpot AlexaFluor 488 (Biotium, San Francisco, California, USA) in PBS for 20 minutes. Sections were mounted and cover slips placed with Prolong Gold Anti‐Fade mounting medium with 4',6‐diamidino‐2‐phenylindole (DAPI, Invitrogen, Carlsbad, California, USA). Negative control for non‐specific binding was done separately with the exception that the primary antibody was omitted.

The Keyence BZ‐X800 all‐in‐one Microscope was used for representative images and microscopic analysis of tissue. For each region of interest assessed, images were taken on three to five identical sections per subject at a representative location, selected using the mouse brain atlas. Bregma used by region were entorhinal cortex and subiculum (‐3.52 to ‐4.04 mm), and CA1 (‐2.92 to ‐3.40 mm). The immunoreactivity (+IR) was quantified in each region using either the BZ‐X800 Analyzer software or ImageJ Analysis Software and reported as +IR pixels/mm^2^. For localization of lipid within lysosomes or neurons, the number of pixels within the lysosomal (LAMP1+), neuronal (MAP2+), or neuronal lysosomes (MAP2+/LAMP1+) region of interest (ROI) was measured using the BZ‐X800 Analyzer software. Pearson's correlation coefficients were calculated for lysosomal lipid using ImageJ™.

### 2.10 Hippocampal‐entorhinal brain slice culture

Hippocampal‐entorhinal brain slice culture (HEBSC) was performed using techniques routinely used in our laboratory.^45^, ^50^ Briefly, sections were prepared from either the P7 or adult (6 mo.) 3x‐Tg‐AD hippocampal‐entorhinal cortex. Initial experiments were done on HEBSC from P7 mice then in HEBSC from adults to ensure developmental differences did not drive findings. Mice were decapitated, brains extracted, and the hippocampal–entorhinal complex dissected in Gey's buffer (Sigma‐Aldrich). Slices were cut transversely at 375 µm using a McIlwain tissue chopper and placed on Millicell culture inserts (Millipore, PICMORG50, up to 13 slices per insert). Slices acclimated in culture media (MEM plus 24 mM HEPES and Hank's salts, 25% horse serum, 5.5 g/L glucose, 2 mM L‐glutamine) in a humified 5% CO_2_ incubator for 7 days, followed by 12% horse serum for four days, and 6% horse serum until the completion of the experiments.

### 2.11 Behavior

#### 2.11.1 Strategy for longitudinal assessments with aging

In order to evaluate the impact of LAL modulation on progressive changes in cognitive function and affect in the AD model, we performed behavioral assays at several different time points in the same set of mice. We limited carry‐over effects from multiple testing by ordering procedures from the least stressful to the most stressful, with extended recovery time between each round of testing.^51^, ^52^ For LAL GT experiments mice were either assessed beginning at 11 months of age (shLAL experiments and LAL GT rescue after chronic alcohol) or from 6‐13 months of age (LAL GT given at 3 mo.) for locomotion (open field), anxiety‐like behavior (center time in open field), startle reflex with non‐associative memory (acoustic startle), spatial learning (Barnes maze and Morris water maze [MWM]), and Pavlovian associative memory (conditioned fear memory).

#### 2.11.2 Open field

Exploratory activity in a novel environment was used to ensure none of the mice had overt motor impairment or were notably hypoactive (a sign of possible health issues) before starting evaluation in the MWM. This was tested by a 1‐hour trial in an open field chamber (41 cm x 41 cm x 30 cm, 120 lux at the edge, 155 lux in the center) crossed by a grid of photobeams (VersaMax system, AccuScan Instruments). Counts were taken of the number of photobeams broken during the 5‐minute intervals, with measures for locomotor activity (total distance traveled) and vertical rearing movements. Time spent in the center region was used as an index of anxiety‐like behavior.

#### 2.11.3 Acoustic startle response with non‐associative memory habituation

Acoustic startle was used to assess auditory function as well as reactivity and memory habituation to an environmental stimulus. Mice were assessed for a reflexive whole‐body flinch (i.e., startle response) after a sudden noise within individual small Plexiglas cylinders within larger, sound‐attenuating chambers (250 lux, San Diego Instruments SR‐Lab system). Each cylinder is placed upon a piezoelectric transducer, which records the force of startle responses. The chambers have a ceiling light, fan, and loudspeaker for the acoustic stimuli. A 5‐minute habituation period precedes the acoustic startle (40 ms; 120 dB) with startle amplitude measured during a 65‐ms sampling window. Startle habituation with aging was measured for each subject by comparing their individual startle magnitude at 8 and 12 months with the response at 6 months. The following equation was used: (Startle_x_—Startle_6mo_)/Startle_6mo_ x 100%.

#### 2.11.4 Barnes maze

The Barnes mazes was used to evaluate spatial learning as we have reported.^53^ The Barnes Maze is a well‐lit (400 lux), circular platform 122 cm in diameter that is elevated 96.5 cm from the floor. The maze contains 40 holes (5 cm in diameter each) along the perimeter. An escape box was placed under one of the holes, and mice underwent one trial per day of up to 5 minutes to learn the location of the escape hole. Each mouse had his own unique escape location, for each of the learning days which continued until each group reached an average of 20 seconds for latency to escape. The experimenter remained blinded to the treatment group.

#### 2.11.5 MWM

The water maze was used to evaluate spatial and reversal learning, swimming ability, and vision. The water maze consisted of a large circular pool (diameter = 122 cm, 320 lux) partially filled with water (45 cm deep, 24° C–26° C), located in a room with numerous visual cues. The procedure involved a visible platform test, acquisition in the hidden platform task, and a test for reversal learning (an index of cognitive flexibility).

*Visible platform test*

Each mouse was given four trials per day, across 2 days, to swim to an escape platform cued by a patterned cylinder extending above the surface of the water. For each trial, the mouse was placed in the pool at one of four possible locations (randomly ordered) and then given 60 seconds to find the visible platform. If the mouse found the platform, the trial ended, and the animal was allowed to remain 10 seconds on the platform before the next trial began. If the platform was not found, the mouse was placed on the platform for 10 seconds and then given the next trial. Measures were taken of latency to find the platform and swimming speed via an automated tracking system (Noldus Ethovision).

*Acquisition and reversal learning in a hidden platform task*

Following the visible platform task, mice were tested for their ability to find a submerged, hidden escape platform (diameter = 12 cm). Each mouse was given four trials per day, with 1 minute per trial, to swim to the hidden platform. The criterion for learning was an average group latency of 15 seconds or less to locate the platform. Mice were tested until the group reached criterion, with a maximum of 9 days of testing. When the group reached criterion (on day 5 in the present study), mice were given a 1‐minute probe trial in the pool with the platform removed. Selective quadrant search was evaluated by measuring percent time in the quadrant where the platform (the target) had been placed during training, versus the opposite quadrant, and number of crosses over the target location where the platform had been placed, versus the corresponding area in the opposite quadrant. Following the acquisition phase, mice were tested for reversal learning, using the same procedure as described above. In this phase, the hidden platform was re‐located to the opposite quadrant in the pool. As before, measures were taken of latency to find the platform. On day 6 of testing, the platform was removed from the pool, and the group was given a probe trial to evaluate reversal learning.

#### 2.11.6 Conditioned fear acquisition, stress reactivity, and memory

This Pavlovian test for associative learning and memory was conducted using a Near‐Infrared image tracking system (120 lux, MED Associates, Burlington, Vermont, USA). On day 1, mice underwent a 7‐minute acquisition session. Mice were first allowed to habituate to the test chamber for 2 minutes. Mice then underwent three pairings of an 80 dB, 30‐second tone (unconditioned stimulus [US]), which was immediately followed by a 2‐second scrambled foot shock (0.4 mA, conditioned stimulus [CS]). The following day, mice were assessed for context‐induced stress reactivity, a post‐traumatic stress disorder (PTSD)‐like phenotype in the original test chamber, with levels of freezing (immobility) measured during a 5‐minute session. On day 3, mice were assessed for cue‐dependent stress reactivity in a modified version of the chamber (plexiglass inserts to alter the walls and floor, and a mild novel odor of 70% ethanol added) with re‐exposure to the 80 dB tone after a 2‐minute habituation period. Two weeks later, contextual, and cue‐related associative memory were assessed on consecutive days.

#### 2.11.7 Power analyses

Power analyses were carried out by the UNC Mouse Behavioral Phenotyping Core, using data from two different mutant lines with established alterations in multiple assays. The analyses indicated that overall optimal sample sizes for behavioral experiments could range from 5 to 11 animals per treatment group, in order to detect significant differences 95% of the time, while sample sizes of 7–14 animals per treatment group would be necessary for the higher criterion for power detection of 99%. For LAL GT given at 3 months, we further optimized our ability to detect significant effects of the LAL modification by using only female mice, which have more robust pathology in this model,^54^ to avoid increased variability from including both sexes

### 2.12 Chromatin immunoprecipitation

Chromatin immunoprecipitation (ChIP) was performed as previously described by our group.^55^ Briefly, *post mortem* human hippocampal tissue from CON and AD individuals was homogenized, cross‐linked with 1.0% methanol‐free formaldehyde, quenched with 1.0 M glycine, lysed with lysis buffer (1.0% [v/v] SDS, 10 mM EDTA, 50 mM Tris‐HCl [pH 8.0]), and chromatin sheared to fragments of < 1000 bp on a Covaris ME220. Input DNA fractions were removed from the sheared chromatin to be processed separately, and the remaining sheared chromatin was incubated overnight at 4°C with an antibody against rabbit FoxO1, rabbit phospho‐Rpb1, or the negative control rabbit immunoglobulin G (IgG). Protein A Dynabeads were added and rotated at 4°C for 1 hour followed by five washes in ChIP wash buffer. Both immunoprecipitated DNA and input DNA were eluted in 10% (w/v) Chelex by boiling at 95°C for 10 minutes followed by centrifugation. ChIP‐enriched DNA was analyzed using qPCR with SSOAdvanced Universal SYBR Green Supermix (Bio‐Rad, Berkeley, California, USA) using primers designed against regions of the *LIPA* gene. The ΔΔCt method was used to determine fold occupancy relative to control and was normalized to the input DNA fraction.

### 2.13 Statistical analyses

The specific statistical test used is noted for each assessment above and were performed in GraphPad Prism. For preplanned orthogonal contrasts, *t‐*tests were used. For age‐matched assessments paired *t‐*tests were employed. One‐way or two‐way analyses of variance (ANOVAs) were used for multiple‐group assessments. Dunnett's or Sidak's post‐tests were used for ANOVAs when appropriate. F and *p* values not included in the figure legends are provided in Table S3. Outliers were detected using the Grubb's test. Studies were not powered specifically to detect sex differences. However, data are presented dissagregated by sex wherever signs of potential sex differences emerged.

## 3 RESULTS

### 3.1 LOAD midlife risk factors promote AD pathology by disrupting neuronal Aβ metabolism

We first assessed the impact of midlife heavy alcohol (i.e., ethanol) and diet‐induced obesity on LOAD pathology in the entorhinal cortex (ENT), hippocampus, subiculum (SUB), and frontal cortex (FCX). The 3xTg‐AD mouse model (APPSwe, tauP301, Psen1^tm1Mpm^) was used since it shows a progressive age‐related increase in pathologic Aβ and tau species, seen first in entorhinal and hippocampal regions, followed by cortex at later ages, similar to LOAD.^21^, ^56^ Mice were treated during midlife to match the LOAD epidemiology, when Aβ pathology is ∼30%–40% of peak levels in 3xTg‐AD. Since mice metabolize ethanol approximately eight times more rapidly than humans^46^; a dose was used that produces average blood alcohol pharmacokinetics similar to those in humans during binge use (AUC: 230.7mMxhour).^47^ Given that females have an increased risk for AD and that female 3xTg‐AD mice having greater pathology than males in some regions,^54^ results were stratified by sex when signs of sex differences emerged or there was a rationale to expect sex differences based on the model. Ethanol increased cellular staining of Aβ1‐42 by ∼55%–60% in the ENT and FCX in both sexes, with higher baseline levels in the female FCX (Figure 1A–D). At this age, only females show amyloid plaques which are found in the SUB. Ethanol increased Aβ plaques by two‐fold (Figure 1E,F). Ethanol also increased p‐tau‐181, a marker for LOAD in humans,^57^ by 40% in CA1 and FCX (Figure 1G–J). These increases occurred without any effect on expression of the human *hAPP* or *MAPT* transgenes in cortex (Figure S1A–C). In WT mice, ethanol likewise increased total amyloid precursor protein (APP) in WT ENT (∼36%), without altering expression of mouse *APP*, suggesting that AD genetics are not required for this effect (Figures 1K–L, S1D). Furthermore, in 3xTg‐AD, ethanol had no effect on levels of cortical presenilin 1 (PSEN1) or BACE1 protein (Figure S1E,F) and had minimal impacts on the major tau‐phosphorylating kinases (pSer9‐GSK3β, pTyr216‐GSK3β, p‐PKA) and the tau phosphatase PP2A (Figure S1G–K). Western diet caused obesity during midlife which increased Aβ1‐42 at 11 months 0.5 to 2.5‐fold in ENT, FCX, and SUB (2.4‐fold) (Figures 1 M–R, S1L). Similar to ethanol, this occurred without any differences in expression of the *hAPP* transgene (Figure S1 M,N) or levels of PSEN1 or BACE1 (Figure S1O–P). Midlife obesity also increased p‐tau181 in CA1 (41%, *p = *0.05, Figure 1S‐,T) with no changes in expression of the *hMAPT* transgene (Figure S1Q), nor tau phosphorylating enzymes p‐PKA, GSK3β, or CDK5 in cortex or hippocampus (Figure S1R–Y).

These findings in rodents were consistent with human *post mortem* ventromedial prefrontal cortex (vm‐PFC/BA25) and hippocampus from individuals with alcohol use disorder (AUD) or moderate‐drinking age‐matched controls. Though none of these individuals had a comorbid diagnosis of AD, AUD brains had higher Braak scores than controls (Figure 1U) and increased levels of p‐tau214 and p‐tau181 in vm‐PFC (Figure 1V,W, ∼50% and 30%, respectively) and hippocampus (Figure 1X,Y, 20%–27%) without increases in total tau. Further, Aβ1‐42 was increased in AUD vm‐PFC (31%, Figure 1Z‐AA), with no changes in levels of APP. Similar to findings in 3xTg‐AD, gene expression of *APP*, *MAPT*, *GSK3*, and *CDK5* were unchanged in AUD hippocampus (Figure S1Z). Tau‐phosphorylating isoforms of GSK3 were increased in AUD BA25 (Figure S1AA‐BB), while levels of Aβ1‐42 producing BACE1 protein were unchanged (Figure S1CC). Together, this indicates that both midlife alcohol and obesity enhance AD pathology without altering Aβ or tau gene expression or levels of Aβ modifying enzymes. This suggests that both of these LOAD risk exposures disrupt the metabolism/degradation of Aβ rather than increasing its production or processing.

### 3.2 Midlife alcohol or obesity disrupt autophagic flux through lysosomal dysfunction

Since the findings above suggested midlife alcohol and obesity LOAD risk exposures disrupt neuronal Aβ metabolism, we assessed the autophago‐lysosomal system, the main site of intraneuronal Aβ degradation,^32^, ^33^ for convergent changes. Both insults were found to uniquely disrupt autophagy. Midlife ethanol increased p62 (∼20%) and reduced the autophagy initiator Beclin (20%) in males and female 3xTg‐AD cortex (Figure 2A,B), while increasing the autophagosome elongation factor LC3‐II in males (Figure 2C, ∼20%). Ethanol further increased the mature lysosome protein LAMP1 with a greater effect in females (Figure 2D), and increased levels phosphorylated mTOR (p‐mTOR, two‐fold, Ser4228) a master regulator of autophagy in both sexes (Figure S2A). These suggest a loss of autophagic flux due to lysosomal dysfunction with a secondary reduction in autophagy initiation (Figure 2E). Diet‐induced midlife obesity also disrupted autophagic flux, though in a different manner than ethanol. Obesity increased p62 (∼33%) and reduced Beclin (∼21%) in both sexes (Figure 2F–G). A main effect of obesity on LC3 was found along with reduced LAMP1 in males (Figure 2H,I) with no changes in p‐mTOR Ser4228 (Figure S2B). This also suggests lysosomal dysfunction, though to a different degree than with alcohol (Figure 2J). In WT mice without AD genetics, ethanol caused a less severe disruption seen with increased P62 in females, reduced Beclin and LC3 in both sexes, and LAMP1 accumulation in females (Figure S2D–G). In 11‐month obese WT mice, no significant changes in P62, Beclin, LC3, or LAMP1 were seen (Figure S2I–L). Thus, there may be interaction between early AD pathology and midlife risk exposures on the autophago‐lysosomal system.

To further assess lysosomal dysfunction, we measured the expression of lysosomal lipases and proteases, regulators of lysosome/autophagosome fusion, and lysosomal structural genes. Both LOAD risk exposures reduced expression of lysosome acidifying v‐ATPases (Figure 2K), and ethanol was found to reduce lysosomal acidification in the ENT of hippocampal‐ENT brain slice cultures (HEBSC, Figure 2L,M). This was seen with reduced levels of TFEB, a master regulator of lysosomal gene expression, with ethanol (Figure 2N) and obesity (Figure 2O). Similar reductions in TFEB and lysosomal v‐ATPases were also seen in WT females with ethanol (Figure S2L,M). We then assessed several genes that facilitate lysosome‐autophagosome fusion and regulate lysosomal structure in 3xTg‐AD mice after ethanol or obesity. Many were unchanged, however, both ethanol and obesity converged with reduced LAL gene expression (*LIPA*, 20‐25%, Supplemental Figure 2C) with similar reductions in LAL protein (10‐20%, Figure 2P‐Q). Of note, both TFEB and LAL were highly expressed in neurons, suggesting a role for dysfunction of neuronal lysosomes (Figure S2N–O). In human AUD brain, autophagic‐lysosomal dysfunction was also seen with reduced LC3 (20%) that was inversely correlated with lifetime alcohol use and severity of AUD using the alcohol use disorders identification test (AUDIT) score (Figure 2R,S). Further, LAL protein was reduced in AUD similar to mouse models in the vm‐PFC (Figure 2T–V) as well as other AD‐associated regions including ENT (39%), CA1 (27%), and SUB (54%) (Figure S2P,Q). Together, this indicates these two common LOAD risk factors disrupt autophago‐lysosomal flux with a loss of LAL.

### 3.3 Lysosomal lipid accumulation is positively associated with extra‐lysosomal Aβ levels

Since LAL is reduced by both midlife alcohol and obesity, we next determined if lysosomal lipid accumulation is associated with Aβ pathology. Total intracellular neutral lipid was increased in both sexes with each of these LOAD risk exposures in FCX and ENT (Figure 3A,B). Greater increases were seen in the ENT, particularly in females, which develops amyloid pathology prior to the FCX in the 3xTg‐AD model and shows increased pathology in females.^54^, ^56^ Notably, significant lipid increases were found within lysosomes in male and female FCX as well as ENT (Figure 3D,E) with increased Pearson's colocalization correlation coefficients (Table S4). In the FCX, cytosolic (i.e., non‐lysosomal) lipid was unchanged in both sexes (Figure 3F). In the ENT, ethanol caused a slight but significant increase in cytosolic lipid, with obesity causing a robust increase (Figure 3G). Of note, similar alterations in lipids were observed in both males and females. Further, lipid accumulation was also found in microglia consistent with a previous report (Figure S3A–C).^58^ Consistent with the findings above, ethanol and obesity increased total amyloid in the cortex, which was seen within neurons, since Aβ plaques were not found in these cortical regions at this age. These increases in amyloid were found outside of lysosomes in the FCX (Figure 3H) and ENT (Figure 3I), though females also showed increases in lysosomal Aβ in ENT. This was associated with reductions in the Aβ lyso:cyto ratios consistent with exclusion of Aβ from neuronal lysosomes (Figure 3J). With both exposures, total amyloid was positively correlated with lysosomal lipid levels in FCX (Figure 3L, *R* = 0.57, ****p < 0.0001). Similarly, neurotoxic Aβ1‐42 was strongly correlated with lysosomal lipid in the ENT (Figure 3M, *R* = 0.52, ***p = 0.003). However, there was no such correlation with cytoplasmic lipid levels with ethanol neither in the FCX nor ENT (Figure 3N,O), indicating that lysosomal, rather than cytosolic, lipid accumulation is associated with Aβ accumulation. High fat diet‐induced obesity, however, increased lipids in lysosomal and cytoplasmic compartments to similar extents likely due to the dietary excess of exogenous lipids, with positive correlations with Aβ in both compartments (Figure S3D,E). WT mice treated with ethanol had increased total (83%) and lysosomal lipid (2.5‐fold) with a strong correlation between total amyloid and total lipid (*R* = 0.7, *p < 0.05, Figure S3F–I), indicating AD genetics are not required for the effect of ethanol. CA1 p‐tau‐181 was also increased by ethanol, with the majority of p‐tau was found outside of lysosomes, and no significant change in the lyso:cyto ratio, suggesting an alternative mechanism (Figure S3J–L). Further, we did not find evidence for ER stress, which can promote lipid droplet formation (Figure S3M). However, both exposures altered the expression of lipid‐droplet coating perilipins (PLINs) and reduced lipid efflux transporters *ABCG1* and *ABCA1* (Figure S3N). In sum, this indicates a strong association between lysosomal lipid and amyloid accumulation.

Neither ethanol nor obesity caused convergent changes in the expression of common upstream regulators of lipid metabolism or production (Figure S3O–Q). However, we and others have reported that proinflammatory microglia activation promotes Aβ accumulation and neurotoxicity.^38^, ^45^, ^59^ Therefore, we investigated if proinflammatory microglia promote NLL. HEBSCs from 3xTg‐AD mice were transfected with a Gi inhibitory DREADD (AAV9.CD68.hM4di) to inhibit proinflammatory signaling as we previously reported.^45^ Inhibition of microglial proinflammatory activation prevented ethanol‐induced increases in intracellular lipid (Figure 3P,Q). This also occurred in vivo using CX3CR1Cre^ERT2^.hM4di‐DIO mice. Ethanol promoted proinflammatory microglial activation with increases in neuronal lipid that was prevented by microglial inhibition (Figure 3R–T). Together, this indicates that proinflammatory microglia promote NLL, which is associated with Aβ pathology. Therefore, we next investigated if the loss of LAL and subsequent NLL contributes to Aβ accumulation.

### 3.4 LAL is lost with aging, with NLL and enhancement of amyloid accumulation

**FIGURE 4:**
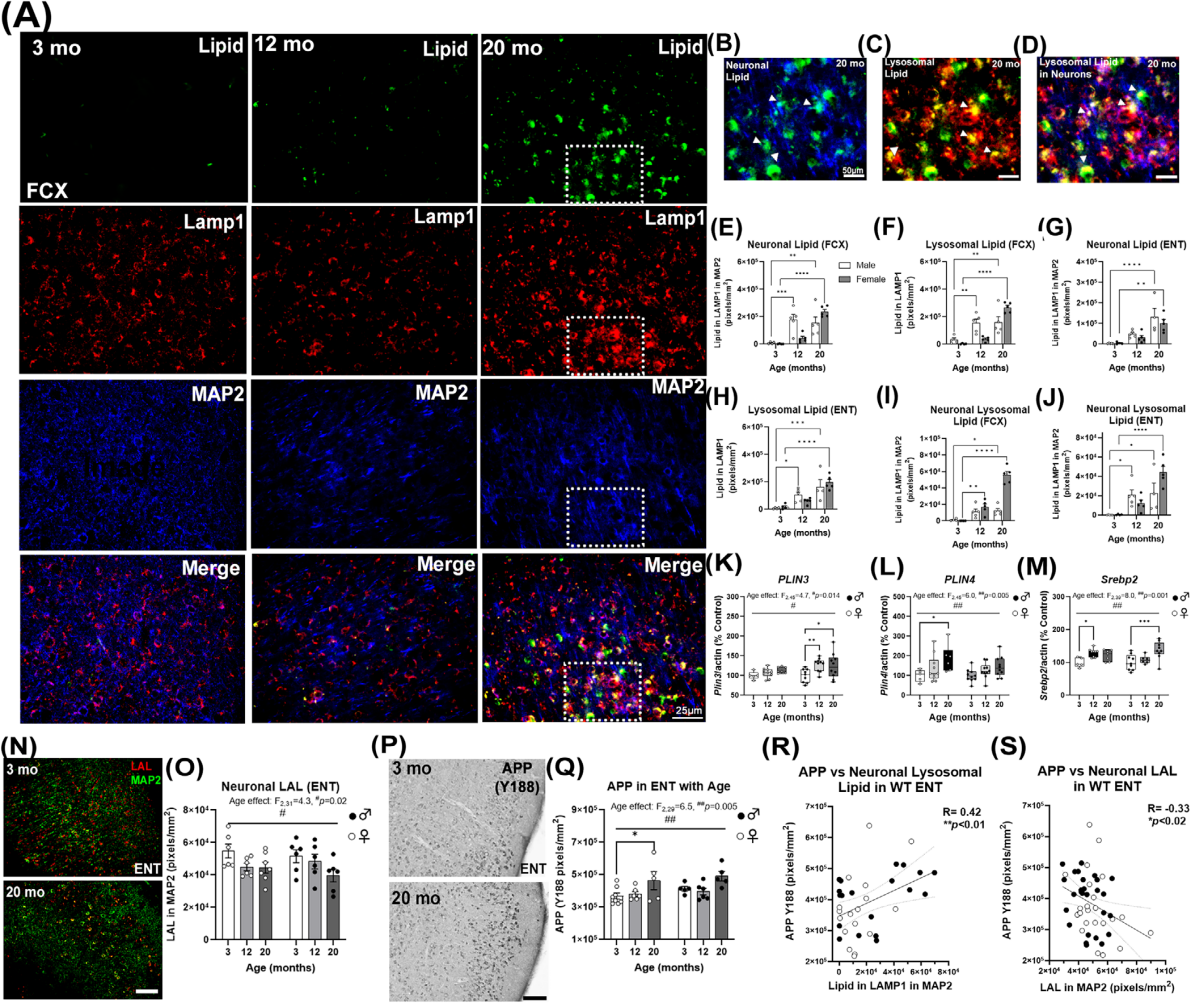
Loss of neuronal LAL and accumulation of neuronal lysosomal lipid with aging in wild‐type mice. (A) Representative images of cytosolic lipid (LipidSpot), lysosomes (LAMP1), and neurons (MAP2) at 3, 12, and 20 months of age in frontal cortex (FCX). Higher magnification images of (B) neuronal lipid, (C) lysosomal lipid, and (D) neuronal lysosomal lipid at 20 months. Profound aging‐related increases in FCX (E) neuronal lipid (F_2,22‐age _= 21.24, *p <* 0.0001) and (F) lysosomal lipid (F_2,22‐age _= 30.64, *p <* 0.0001), as well as ENT (G) neuronal lipid (F_2,22‐age _= 20.2, *p <* 0.0001) and (H) lysosomal lipid (F_2,22‐age _= 23.7, *p <* 0.0001). Increased NLL was seen in (I) FCX (F_2,22‐age _= 53.1, *p <* 0.0001) and (J) ENT (F_2,22‐age _= 18.1, *p <* 0.0001) from 3 to 20 months. Two‐way ANOVAs, *p < 0.05, **p < 0.01, ***p < 0.001, ****p < 0.0001, Dunnett's post‐test. Increased gene expression of (K) *PLIN3*, Age effect F_2,45 _= 4.7, #*p = *0.014, and (L) *PLIN4*, F_2,45 _= 6.0, ##*p *= 0.005 and (M) *Srepb2* expression increases with age. F_2,39 _= 8.0, ##*p *= 0.001. *p < 0.05, **p < 0.01, ***p < 0.001, Dunnett's post‐test. (N, O) Loss of neuronal LAL (LAL‐red within MAP2/green) in WT ENT (∼30%) with age in both sexes. F_2,31 _= 4.3, #*p *= 0.02. (P‐Q) An age‐related increase in total amyloid precursor protein (APP, ∼30%) in ENT from 3 to 20 months. F_2,29 _= 6.2, ##*p *= 0.005. *p < 0.05, Dunnett's post‐test. (R) ENT APP was positively correlated with neuronal lysosomal lipid in WT ENT. (S) Negative correlation between APP and neuronal LAL. Males, open circles; females, filled circles.

LOAD pathology emerges in humans and 3xTg‐AD mice with aging, suggesting that the loss of underlying resilience mechanisms with age is key for the emergence of AD. To determine if LAL loss is an age‐related risk factor for LOAD, we first assessed WT mice across normal aging. At 3 months, very little intracellular lipid was seen (Figure 4A). However, by 20 months, profound increases in lipid were found in neurons, lysosomes, and neuronal lysosomes with some differences in trajectory between sexes (Figure 4B–D). In FCX, intracellular lipid increased 280‐fold from 3 to 20 months in females, with males reaching a stable 19‐fold increase by 12 months (Figure S4A). Neuronal and lysosomal lipid also increased greatly in male and female FCX (Figure 4E,F) with similar changes seen in ENT for total lipid (Figure S4B), neuronal lipid, and lysosomal lipid (Figure 4G,H, Table S4). No differences were seen in neuronal lysosomes (Figure S4C). However, robust increases in NLL were found, that reached higher levels in females (Figure 4I,J). Notably, the expression of *PLIN3* and *PLIN4* increased in both sexes (Figure 4K,L) as did the lipogenic factor *Srebp2* (Figure 4M). In general, females, which show increased risk for LOAD, had a more active lipid regulatory profile than males (Figure S4H,I). This includes increases in *LXRα* and metabolic genes *LCAD and MCAD*. In ENT, total and neuronal LAL declined with age in both sexes (Figures 4N,O and S4D), while in FCX, a main effect of age on LAL was found with females already having lower levels at 3 months than males (Figure S4E, F_2,29 _= 20.64, *p <* 0.001). Age‐related reductions in neuronal LAL in FCX were less robust than in ENT, though males showed a slight decline from 3 to 12 months (Figure S4F). At 12 and 20 months, NLL was inversely correlated with neuronal LAL (Figure S4G, *R* = ‐0.38, *p < 0.05). In WT mice, amyloid does not normally form aggregates. However, steady‐state levels of neuronal APP are maintained through endocytosis and degradation via the endosomal lysosomal system.^27^, ^28^, ^29^, ^30^, ^31^, ^32^, ^33^ Since findings in AD and WT mice were consistent with lysosomal dysfunction (Figures 2 andS2), we assessed APP in WT mice with age. Total APP increased in the WT ENT from 3 to 20 months (25%, Figure 4P,Q) was positively correlated with NLL (Figure 4R, *R* = 0.45, **p < 0.01), inversely correlated with neuronal LAL (Figure 4S, *R* = ‐0.33, *p < 0.02), and appeared to lag slightly behind LAL loss. This indicates that, in normal aging, NLL increases and is associated with APP levels, similar to findings in AD mice. Therefore, we hypothesized next that LAL loss and the subsequent lysosomal dysfunction disrupts the steady state balance of neuronal amyloid.

**FIGURE 5:**
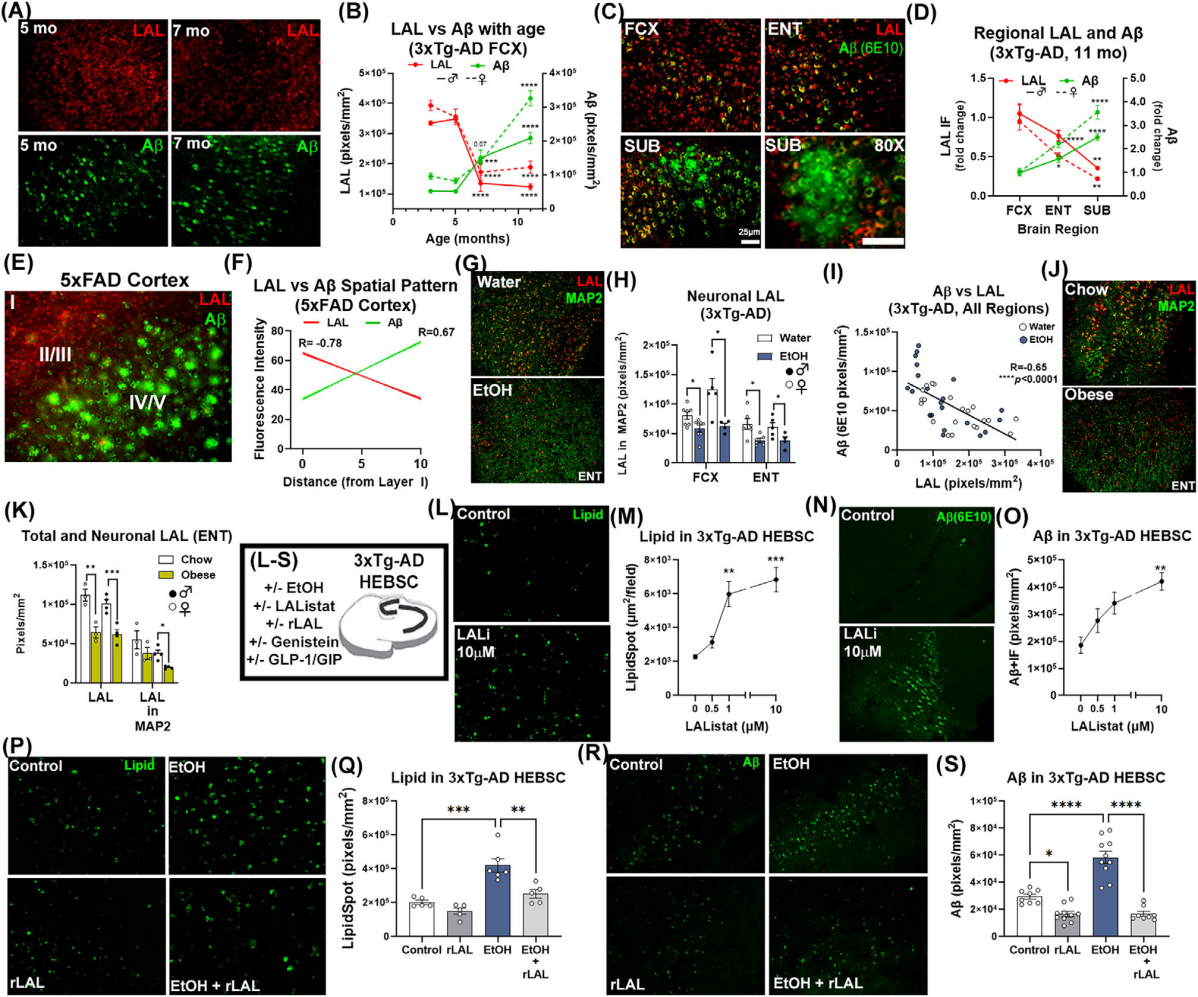
Loss of neuronal LAL promotes Aβ accumulation. (A‐B) Loss of LAL precedes Aβ accumulation in 3xTg‐AD FCX in males (Age effect LAL: F_3,18 _= 88.3, *p <* 0.0001, Aβ: F_3,16 _= 36.6, *p <* 0.0001) and females (LAL: F_3,15 _= 25.5, *p <* 0.0001, Aβ: F_3,14 _= 73.3, *p <* 0.0001). ***p < 0.001, ****p < 0.0001 Sidak's post‐test. (C, D) 3xTg‐AD brain regions where FCX had the highest levels of LAL and the lowest levels of Aβ, ENT had intermediate levels of both, and subiculum (SUB) had Aβ plaques and low levels of LAL. Region effect: F_2,72 _= 21.6, *p <* 0.0001. **p < 0.01, ***p < 0.001, ****p < 0.0001 vs. FCX, Dunnett's post‐test. (E) Representative image of 5xFAD cortex showing inverse spatial association of LAL and Aβ across cortical layers at 3 and 6 months of age. (F) Averages of fluorescent intensity profiles across cortical layers for LAL and Aβ showing inverse relationship (****p < 0.0001 for each). (G) Representative images of reduced neuronal LAL with ethanol. (H) Ethanol reduced neuronal LAL in both FCX and ENT in males and females. *p < 0.05, *t*‐test. (I) A strong negative correlation between LAL and Aβ1‐42 was found across all subjects in ethanol experiments. R = ‐0.65, *p <* 0.0001. (J) Representative images of reduced neuronal LAL with obesity. (K) Obesity reduced by total and neuronal LAL in the ENT of males (F_1,8 _= 14.07, *p = *0.006) and females (F_1,12 _= 39.2, *p <* 0.0001). (L‐S) Ex vivo studies using hippocampal‐entorhinal brain slice cultures (HEBSC) from adult 3xTg‐AD mice found a causal relationship between LAL activity and Aβ. LAL inhibitor LAListat (LALi) caused a dose‐dependent increase in (L, M) cytosolic lipid as well as (N) Aβ in ENT region of HEBSC. Recombinant LAL (5 µg/mL) blocked ethanol‐induced increases in (P‐Q) cytosolic lipid and (R, S) Aβ. **p < 0.01, ***p < 0.0001, one‐way ANOVA, Tukey's multiple comparisons test.

To investigate a role for LAL loss in the enhancement of Aβ pathology, we first assessed the temporal relationship between LAL and Aβ in AD mice. This revealed that LAL loss with age preceded the age‐related increase in Aβ in the 3xTg‐AD mouse frontal cortex (Figure 5A,B). Further, within individual animals, regions with the highest levels of Aβ pathology (e.g., SUB) had the lowest levels of LAL, while regions with lower Aβ (e.g., FCX) had the higher levels of LAL (Figure 5C,D, 11 mo.). A similar association was found in the 5xFAD mouse, where LAL and Aβ fluorescent intensities were inversely related across frontal cortical layers (Figures 5E,F, S5A). Midlife ethanol reduced total and neuronal LAL across regions with strong negative correlations between total Aβ and LAL (Figures 5G–I, 5B). Midlife obesity likewise reduced total and neuronal LAL with a strong negative correlation between neuronal LAL and Aβ (Figures 5J,K, 5C). To determine if loss of LAL activity causes Aβ accumulation, we employed HEBSCs from 3xTg‐AD mice (Figure 5L‐S). Inhibition of LAL activity with LAListat (LALi) caused an expected concentration‐dependent increase in cytosolic lipid (Figure 5L,M). Strikingly, this resulted in a robust concentration‐dependent increase in Aβ in the ENT, directly implicating the loss of LAL activity with Aβ accumulation (Figure 5N,O). Similar to findings in vivo, ethanol caused an increase in cytosolic lipid (Figure 5P). Neither induction of a major lysosomal transcription factor TFEB with genistein nor agonism of GLP‐1/GIP signaling with DA4‐JC abolished the ethanol‐induced increases in lipid (Figure S5D–G). However, addition of recombinant LAL (rLAL) blocked the two‐fold increase in cytosolic lipid caused by ethanol (Figure 5P,Q). Further, rLAL reduced baseline Aβ levels by 50%, and abolished the two‐fold increase in Aβ caused by ethanol (Figure 5R,S), indicating that LAL supplementation reduces Aβ accumulation. Together, this indicates that LAL loss precedes Aβ accumulation with age, LAL activity is inversely associated with Aβ levels ex vivo, and LAL levels are inversely correlated with Aβ in vivo.

### 3.5 LAL GT improves AD neuropathology and cognition

**FIGURE 6:**
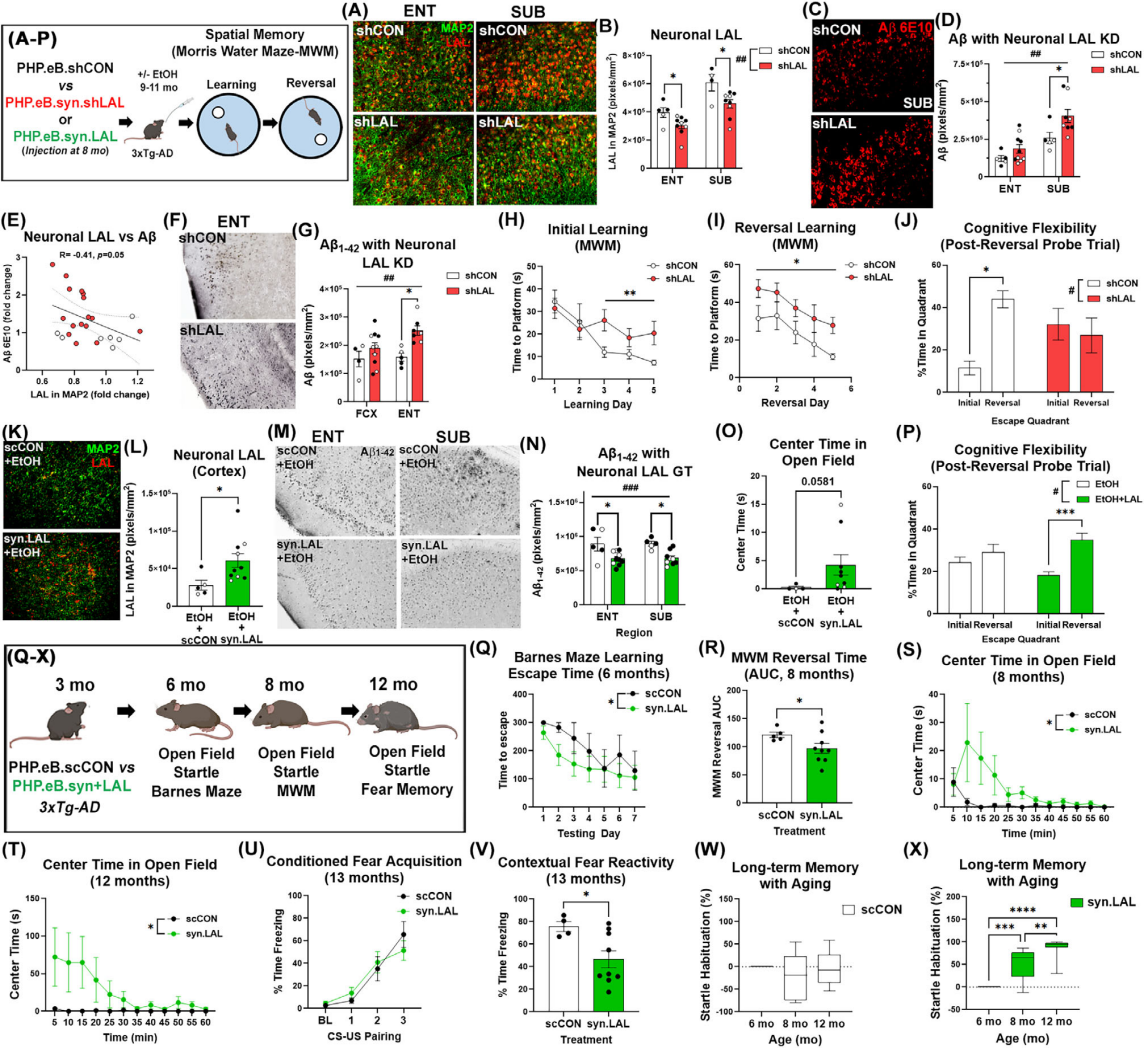
Neuronal LAL regulates Aβ pathology and cognition in vivo. (A‐P) 3xTg‐AD mice received either scrambled RNA control PHP.eB.scRNA, PHP.eB.syn1.shLAL LAL knockdown (KD), or PHP.eB.syn1.LAL.WPRE gene therapy (GT) at 8 months ± ethanol from 9 to 11 months followed by behavioral testing and histological assessment at 13 months. Sexes combined due to lack of difference. Open circles‐males, filled circles‐females. (A, B) Neuronal LAL KD persistently reduced neuronal LAL in ENT and SUB at 13 months (F_1,23 _= 21.18, *##p *< 0.01, *p < 0.05, Sidak's post‐test) and (C, D) increased total amyloid (6E10), 2‐way ANOVA F_1,24 _= 8.3, *##p <* 0.01, *p < 0.05, Sidak's post‐test. (E) Neuronal LAL and amyloid were inversely correlated. (F, G) Neuronal LAL KD increased Aβ1‐42 across FCX and ENT F_1,22 _= 9.5, *##p = *0.005. *p < 0.05, Sidak's post‐test. Neuronal LAL KD impaired (H) learning in the Morris water maze (MWM, F_1,13 _= 5.72, *p < 0.05, repeated measures ANOVA), as well as (I) reversal learning (F_1,11 _= 5.2, *p < 0.05), and (J) cognitive flexibility in the post‐reversal learning probe trial (F_1,24 _= 5.77, treatment × quadrant, #*p <* 0.05, *p < 0.05, Sidak's). (K, L) PHP.eB.syn1.LAL GT given at 8 mo. persistently increased neuronal LAL in the 13 mo. cortex, (M, N) reduced Aβ1‐42 in ENT and SUB of EtOH‐treated mice (F_1,25 _= 18.52, *###p <* 0.001, *p < 0.05, Sidak's), (O) reduced anxiety‐like behavior (thigmotaxis), and (P) improved cognitive flexibility (F_1,24 _= 4.39, treatment x quadrant #*p <* 0.05, ***p < 0.001, Sidak's). (Q‐X) Mice received PHP.eB.scRNA or PHP.eB.syn1.LAL GT at 3 months with cognition and affect assessed with aging. N = 5 scCON, 10 syn.LAL, females. At 6 months, LAL GT (Q) improved learning in the Barnes maze. Two‐way ANOVA treatment effect F_1,91 _= 4.460, *p = 0.037. At 8 months, LAL GT persistently (R) improved cognitive flexibility (reversal learning escape time area under the curve‐AUC, *p < 0.05 Welch's *t‐*test) and (S) reduced anxiety‐like behavior (F_1,155 _= 5.4, *p = 0.02). At 12 months a persistent improvement in affect was seen with reduced (T) anxiety‐like behavior (F_1,155 _= 8.1, **p = 0.005) and (U, V) contextual fear reactivity. (U) LAL GT had no effect on hearing (80 dB) and conditioned fear acquisition but (V) reduced context‐induced stress reactivity 24 hour later. *p < 0.05, *t‐*test. (W‐X) LAL GT improved long‐term memory. (W) 3xTg‐AD mice showed a deficit in long‐term non‐associative memory formation as assessed by habituation of the acoustic startle response at 6, 8, and 12 months of age. (X) LAL GT restored long‐term memory formation with robust habituation to the startle response observed. One‐way ANOVA with mixed‐effects model adjusted for repeated measures F_1.4_,_12.3 _= 37.4, *p <* 0.00001. **p < 0.01, ***p < 0.001, ****p < 0.0001 Dunnett's post‐test.

Next, to find if neuronal LAL impacts Aβ pathology and cognitive function in vivo, 3xTg‐AD mice were given either *PHP.eB.syn.shLAL* or *PHP.eB.syn.LAL.WPRE* viruses that cross the mouse blood–brain barrier (BBB)^60^ to either knock down or over express LAL, respectively. As expected, *PHP.eB.syn.shLAL* reduced neuronal LAL in the ENT and SUB (Figure 6A,B), resulting in increases in total as well as neuronal lipid (Figure S6A–D). Similar to findings ex vivo, neuronal LAL knockdown caused an increase in amyloid (Figure 6C,D) that was both positively correlated with neuronal lipid (Figure S6E, *R* = 0.69, **p < 0.0001) and inversely correlated with neuronal LAL across all regions assessed (Figure 6E, *R* = ‐0.41, *p = *0.05). Neuronal LAL KD also increased neurotoxic Aβ1‐42 across the frontal and entorhinal cortices (Figure 6F,G). Further, mice with neuronal LAL knockdown showed deficits in learning and memory in the MWM (Figure 5H) as well as impaired reversal learning and cognitive flexibility consistent with a worsening of AD progression (Figure 5I,J). Since this indicates that neuronal LAL knock‐down worsens AD pathology, we next determined if LAL neuronal GT could reduce AD pathology, cognitive decline, and prevent its enhancement by midlife ethanol.

First, mice received either scrambled RNA control *PHP.eB.scCON* or *PHP.eB.syn.LAL.WPRE* GT injection at 8 months, followed by chronic ethanol. LAL GT increased caused robust increases in LAL gene expression with increases in neuronal LAL protein (approximately two‐fold) in the cortex and hippocampus (Figures 6K, Figure S6F–H). The PHP.eB.syn1 viral GT showed neuronal tropism with no significant changes in microglial or astrocytic LAL (Figure S6I–L). LAL GT resulted in reductions in cytosolic, lysosomal, and neuronal lipid as well as a reduction in non‐lysosomal lipid in the ENT (Figure S6M–R). Since LAL is only active in an acidic pH of the lysosome (< 4.5) and not the cytosol, this suggests improving lysosomal lipid metabolism might have added benefits to the overall cellular lipid burden due to the constant movement of lipids between the intracellular compartments.^61^ LAL GT blunted the enhancement of AD pathology induced by ethanol seen with reduced Aβ1‐42 in the ENT and subiculum (Figure 6M,N). This resulted in reduced anxiety‐like behavior (center time in the open field, Figure 6O) and improved cognitive flexibility in the MWM (Figure 6P). Given this improvement with midlife alcohol and the age‐related loss of LAL, we next assessed if LAL GT could improve cognitive function and affect in AD mice as they age.

3xTg‐AD mice received *PHP.eB.scCON* or *PHP.eB.syn.LAL.WPRE* GT at 3 months, 2 months prior to the notable age‐related decline in LAL (Figure 5B), to allow for maximum LAL gene expression prior to the emergence of AD pathology. To assess for effects of LAL GT on peripheral metabolism, body weights were measured. There were no differences in body weight between control and LAL GT mice at the time of sacrifice. Behavioral assessments were performed longitudinally 3, 5, and 10 months after viral injection (i.e., 6, 8, and 13 months of age). Since AD features both cognitive (memory, behavioral flexibility, and non‐associative memory) and affective symptomatology (anxiety and mood dysfunction), both behavioral domains were assessed. LAL GT improved cognitive function at 6 months of age, seen by improved spatial learning in the Barnes maze (Figure 6Q). A trend toward a slight reduction in total locomotion and hints of reduced anxiety‐like behavior (thigmotaxis) were also seen in the open field (Figure S7A,B). Improved cognitive flexibility was seen at 8 months of age, evidenced by a specific improvement in reversal learning escape latency in the MWM (Figures 6R, S7C,D). An improvement in affect was also found at 8 months, as LAL GT reduced anxiety‐like behavior (Figure 6S) without altering total locomotion (Figure S7E). Persistent improvements in affect were seen at 13 months of age, seen by reduced anxiety‐like behavior and contextual fear reactivity (Figures 6T–V, S7F). Both groups equally acquired conditioned fear association with the 80 dB cue, indicating effective hearing and associative learning (Figure 6U). However, LAL GT reduced acute contextual, but not cue‐induced, fear reactivity (Figures 6V, Figure S7G). This indicates improved affect at 13 months as LAL GT did not impact associative fear memory 2 weeks later (Figure S7H). LAL GT further improved long‐term memory. Control 3xTg‐AD mice had deficits in long‐term non‐associative memory, seen by a lack of long‐term habituation of the acoustic startle response at 8 and 12 months (Figures 6W, S7J).^62^, ^63^ However, LAL GT improved long‐term memory formation and retrieval, causing robust habituation of the acoustic startle response (Figures 6X, S7K). This protection by LAL GT occurred without significant effects on the expression of lipid transporters or cytosolic lipases (Figure S7K). However, LAL GT did increase lysosomal localization of tau, suggesting a potential improvement in tau clearance (Figure S7L). Thus, LAL GT causes long‐lasting improvements in cognition and affect.

### 3.6 LAL is lost with aging in human brain with greater reductions in LOAD

**FIGURE 7:**
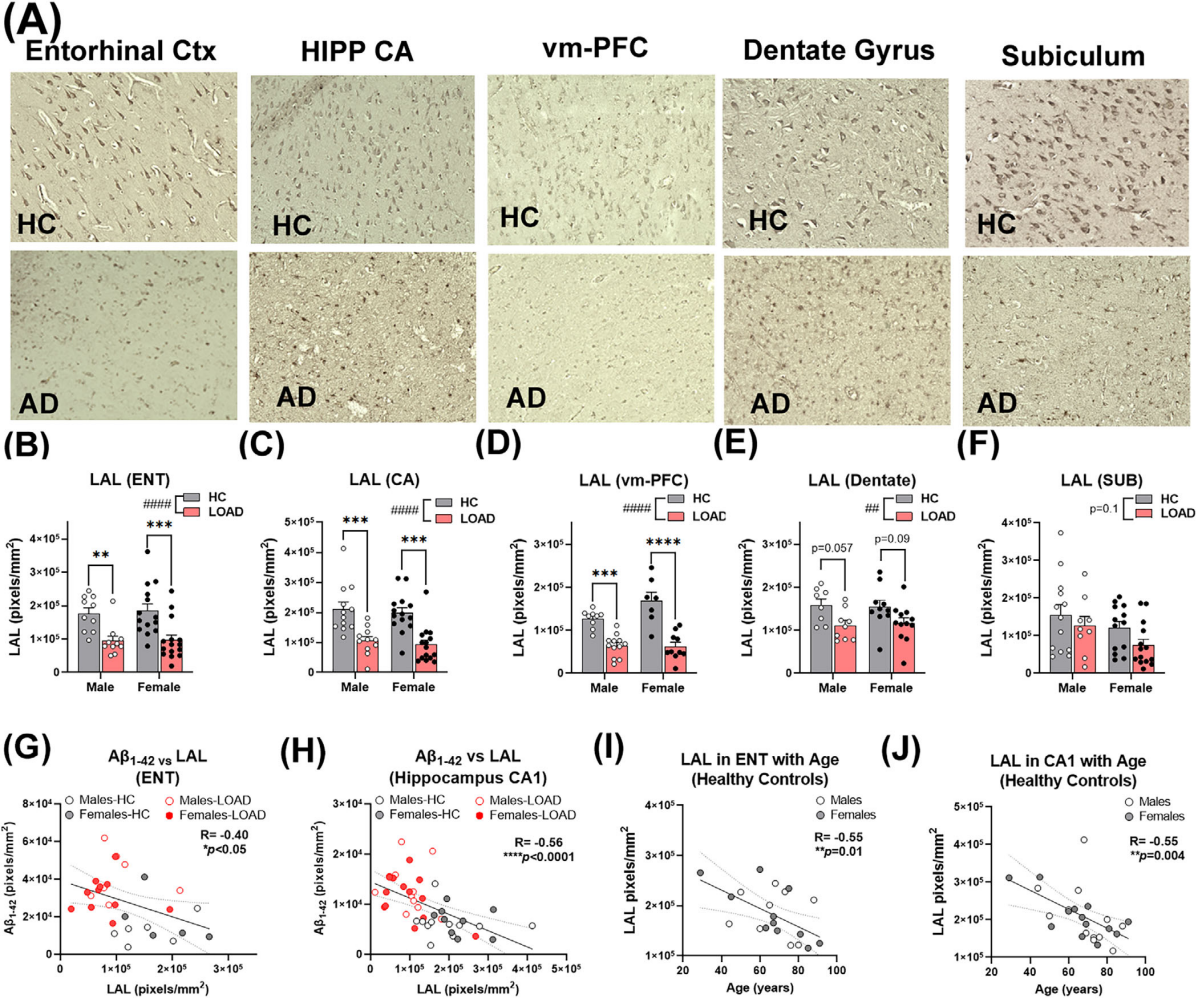
Loss of LAL in humans with LOAD and across normal aging. (A) LAL IHC finding a profound loss of LAL in human LOAD. In healthy control (HC) subjects, LAL was seen in cells with neuronal morphology, which was reduced in LOAD. (B) 47% loss of LAL in ENT (Treatment: F_1,45 _= 22.82, ####*p <* 0.0001), a (C) 52% loss in CA1 (treatment: F_1,48 _= 34.03, ####*p <* 0.0001), a (D) ∼56% loss on the vm‐PFC (Treatment: F_1,34 _= 60.0, ####*p <* 0.0001), a (E) 27% loss in the dentate gyrus (Treatment: F_1,35 _= 9.4, ##*p <* 0.01), and a (F) trend toward a reduction in subiculum (Treatment: F_1,45 _= 2.78, *p *= 0.1). **p < 0.01, ***p < 0.001, ****p < 0.0001, Sidak's post‐test. Open circles‐males filled circles‐females. Strong negative correlations between LAL and Aβ1‐42 staining were seen across all subjects in (G) ENT (*R* = ‐0.4, *p = 0.05) and (H) CA1 (*R* = ‐0.55, ****p < 0.0001). In healthy control subjects, LAL declined with age in (I) ENT and (J) CA1.

To determine if LAL loss occurs in human LOAD and generalizes beyond midlife obesity and alcohol exposures, we measured LAL in brain from LOAD subjects and age‐matched healthy control donors (HC) with no history of AUD. LAL was found in neurons across brain regions (Figure 7A, top). However, in human LOAD, LAL levels were much lower, and the clear neuronal morphology was lost (Figure 7A, bottom). Robust reductions in LAL were found in the ENT (Figure 7B, 47%,), CA (Figure 7C, 70%), vm‐PFC (Figure 7D, 62%), dentate (Figure 7E, 27%), and subiculum (Figure 7F, 56%). Both male and female LOAD subjects showed similar levels of LAL and effect sizes in ENT, CA, and vm‐PFC. In LOAD, Aβ1‐42 was increased in these regions as expected (Figure S8A‐H) with strong negative correlations between LAL and Aβ1‐42 across subjects in CA1 (Figure 7G, *R* = ‐0.60, ****p < 0.0001) and ENT (Figure 7H, *R* = ‐0.5, **p < 0.0015), similar to findings in mice. Further, LAL protein declined with age in healthy subjects in CA1 (Figure 7I, *R* = ‐0.55, **p < 0.004) and ENT (Figure 7J, *R* = ‐0.55, **p < 0.01), though reductions in LOAD were much greater. We then measured LAL gene expression and found a trend toward a reduced expression of the LAL gene (*LIPA)* in LOAD compared to age‐matched controls (Figure S8I), consistent with findings in rodents. Promoter occupancy of its main transcription factor, FOXO1, showed a trend toward an increase as measured by ChIP, though total FOXO1 levels were unchanged (Figure S8J,K). Therefore, we measured the progression of the active RNA polymerase II (p‐RBP1) across the gene body by ChIP (Figure S8L). Binding of p‐RBP1 was increased from the promoter region to exon 3, particularly in females, with suggestions of reduced localization at later exons compared to HCs (Figure S8M). Further, in HCs, though *LIPA* gene expression was positively correlated with p‐RBP1 promoter occupancy as expected (Figure S8N, *R* = 0.64, *p < 0.05), in LOAD no such association was found (Figure S8O). Together, these studies find that LAL protein is lost with aging in human brain with greater losses in LOAD, similar to findings in rodents, with altered the localization of RNA polymerase II across the LAL gene as determined by ChIP‐PCR.

## 4 DISCUSSION

Novel therapeutic targets are needed for AD prevention and treatment. There is ongoing investigation into the early drivers of AD pathogenesis. The emergence of intraneuronal Aβ and extracellular plaque formation is subsequently followed by tau pathology.^5^ However, underlying cellular mechanisms that cross multiple AD behavioral and genetic risk factors, which can be targeted therapeutically need to be defined. Genome‐wide association studies (GWAS) and transcriptomic studies have suggested roles for lipid metabolism and lysosomal dysfunction in AD pathogenesis, though the precise mechanisms in these systems that play a role in AD pathogenesis have been unclear.^6^, ^7^, ^8^, ^10^, ^11^, ^12^, ^16^, ^17^

By comparing two distinct midlife LOAD risk factors, we found that the loss of neuronal LAL precedes and promotes Aβ pathology and cognitive deficits. LAL loss promotes the accumulation of lipid in neuronal lysosomes, which can lead to lysosomal dysfunction. Evidence for this was found in vivo (AD and WT mice), ex vivo (brain slice cultures), and in *post mortem* human LOAD brain. Increased NLL was associated with reduced localization of Aβ to lysosomes, which could be due to a disruption in endo‐lysosomal fusion and reduced lysosomal efficiency, as supported by our observed reduction in lysosomal acidification and the downregulation of vATPases. This would be consistent with recent work finding a reduction of auto‐lysosomal acidification precedes intraneuronal Aβ accumulation and subsequent plaque formation.^12^ However, defects in trafficking to lysosomes or other endo‐lysosomal dysfunction might also contribute and cannot be ruled out. Interestingly, genistein, an agonist of the master lysosomal regulator TFEB, did not fully prevent lipid accumulation. However, LAL supplementation and LAL GT normalized both lysosomal lipid and Aβ levels ex vivo and in vivo. This suggests that normalizing neuronal lysosomal acidification alone may not be sufficient to prevent disease progression, but that enhancing lysosomal lipid degradation may be beneficial. Neuronal knock‐down of LAL in AD mice promoted Aβ accumulation and caused deficits in memory and cognitive flexibility, while LAL GT blunted increases in Aβ pathology and improved spatial memory, cognitive flexibility, affect, and long‐term non‐associative memory. The loss of LAL in human *post mortem* LOAD subjects, who did not have significant alcohol use or obesity, suggests this is a feature of LOAD pathogenesis that extends beyond these two tested risk factors (Figure S9).

Aging is requisite for the development of LOAD. Likewise, though the 3xTg‐AD model features lifelong expression of human familial AD transgenes, pathology emerges slowly with aging. This work finds that the loss of LAL represents the loss of a key resilience mechanism against Aβ accumulation earlier in life. This was supported by findings in WT mice and healthy human brain with aging. NLL increased greatly with age in WT mice along with a loss of LAL and corresponding APP accumulation in the ENT, which parallelled findings in healthy human brain with aging. This suggests that LAL loss with a subsequent increase in NLL is an age‐related phenomenon that produces a neuro‐environment that is vulnerable to LOAD. In human LOAD hippocampus, the loss of LAL protein was accompanied by altered localization of the RNA Poll II and a loss of the positive correlation between LAL gene expression and Poll II promoter binding seen in healthy controls. There was an apparent increase in Poll II binding at the LAL promoter region and early exons, particularly in females, with a possible reduction in Poll II binding at the latter exons. This suggests transcriptional repression might contribute to LAL loss in AD, with potential mechanistic differences between males and females. ChIP‐based approaches do not elucidate the transcriptional activity of the DNA‐bound RNA polymerase.^64^ Therefore, future studies using more robust approaches such as GRO‐seq or NET‐seq are needed to determine definitively if a mechanism such as polymerase pausing is occurring. Pol II pausing was reported with aging in the liver^65^ but has not yet been implicated in AD, which is of significant interest. Nonetheless, LAL GT may represent a promising therapeutic approach, since it can increase LAL in a cell‐type specific manner and would circumvent any potential DNA structural changes, Poll II pausing, or transcriptional alterations in the host genome. LAL GT caused long lasting cognitive and affective benefits, both with aging and after midlife alcohol exposure in AD mice. Ongoing studies are being performed to define the optimal timing and regimen for LAL neuronal GT as well as potential toxicity to determine its utility for potential clinical trials.

In addition to reducing LAL, both alcohol and obesity caused additional disruptions to cellular lipid management. This includes decreased expression of lipid efflux transporters (*ABCG1* and *ABCA1*), which could further promote intracellular lipid accumulation. Polymorphisms in *ABCA1* and other lipid efflux transporters such as *ABCA2* and *ABCA7* are associated with increased risk for LOAD.^16^, ^17^ Further, both alcohol and obesity altered expression of lipid coating *PLIN* proteins in a manner consistent with reduced lysosomal degradation of lipids. Expression of *PLIN4*, which can prevent lysosomal degradation of cytosolic lipid droplets,^66^ was increased with age in WT mice and by both ethanol and obesity in 3xTg‐AD mice, whereas *PLIN2* and *PLIN3*, which promote lysosomal degradation,^67^ were reduced. Together, this suggests that reductions in lipid efflux in addition to deficient lipophagy could ultimately contribute to increases in NLL and the subsequent reduced degradation of intraneuronal amyloid. This opens the opportunity for additional strategies to normalize neuronal lipid levels to attempt to counteract AD pathological progression.

We also found a novel interaction between neuroimmune activation and neuronal lipid metabolism. Several studies have implicated neuroimmune signaling in AD pathogenesis.^68^, ^69^, ^70^ Microglia become increasingly polarized to a proinflammatory state with aging and AD.^58^, ^71^ We found that proinflammatory microglia can directly promote neuronal lipid accumulation. Chemogenetic inhibition of microglia, which we and others have used to prevent proinflammatory microglial activation in various settings,^45^, ^50^, ^72^, ^73^ prevented increases in NLL. Other studies have identified lipid laden microglia with aging and neuropathology,^58^ and a recent study found that neurons can transfer lipid to microglia promoting their dysfunction in the setting of tauopathy.^74^ Our current work implicates microglia as regulators of neuronal lipid metabolism and suggests aged microglia might promote LOAD pathology by causing neuronal lipid dysfunction. Ongoing and future studies will investigate the mechanisms by which microglia regulate neuronal lipid metabolism and the bidirectional nature of this interaction. Unveiling the interaction between neuroimmune and neurometabolic systems could identify novel aspects of AD pathology as well as future therapeutic targets for a variety of neurological conditions.

It is important to note the limitations of this study. First, though the 3xTg‐AD and 5xFAD models provide insight into mechanisms of AD progression, they are models of early‐onset AD (EOAD) rather than LOAD. Though we also find LAL loss in human LOAD brain, there may be differences in the impact of LAL loss in the progression of LOAD versus EOAD. Future studies will employ the newly developed LOAD models from the MODEL‐AD2 consortium to determine if LAL GT is effective in these models. The use of PHP.eB vectors has some limitations such as the potential for expression in the periphery. Though the viruses showed neuronal tropism, transgenic models using neuron‐specific, inducible knockdown and rescue of LAL would more definitively establish its role within neurons without confounds associated with viral delivery. Off target peripheral expression could impact peripheral metabolism. Body weights did not differ between control and LAL GT groups, suggesting this does not contribute; however, more subtle occult changes in the periphery could occur. Nevertheless, the PHP.eB LAL neuronal GT approach, which improved cognition and affect, lays important groundwork for ongoing and future translational studies. Though highly BBB permeant in several mouse strains, PHP.eB vectors do not readily cross the primate, and presumably human, BBB due to the lack of the LY6A transporter.^75^ Thus, our ongoing efforts to translate LAL GT to humans involve intrathecal injection of the AAV9 serotype, which provides robust neuronal transduction throughout the brain, evades anti‐AAV‐neutralizing antibodies and are currently in use in the clinic for other central nervous system (CNS) conditions. Lastly, since the 3xTg‐AD strain was on the B6/129 background and C57BL6 WT mice were used, measures such as the magnitude or trajectory of LAL loss may not completely align. Beyond amyloid, tau plays a critical role in AD symptomatology.^5^ We focused mainly on the relationship between LAL and Aβ, however pathologic tau species are also be degraded by the lysosome. Our finding that LAL GT increases localization of tau to lysosomes suggests tau maintenance might also be improved. Future studies should investigate the role of LAL on tau degradation in models of AD and tauopathy.

In summary, this work finds that the loss of LAL is an important feature of AD pathogenesis. Together, it finds that an age‐related loss in neuronal LAL in combination with midlife LOAD risk factors, or perhaps other neuroimmune or metabolic LOAD risks, can combine to contribute to the progression of AD pathology. It also identifies LAL GT as a promising new approach to prevent and/or treat AD neuropathology and symptomatology.

## CONFLICT OF INTEREST STATEMENT

The first and senior author (Alexandra M. Barnett and Leon G. Coleman Jr) are inventors on patent PCT/US2024/021194, International Publication Number WO 2024/197271 A1 entitled: Targeting lysosomal lipid in Alzheimer's disease. The remaining authors (Lamar Dawkins, Jian Zou, Elizabeth M. McNair, Viktoriya D. Nikolova, Sheryl S. Moy, Greg T. Sutherland, Julia Stevens, Meagan Colie, Kemi Katemboh, Hope Kellner, Katherine Ho, Corina Damian, Sagan DeCastro, Ryan P. Vetreno) have no disclosures to report. Author disclosures are available in the Supporting Information.

## Supporting information



Supporting Information

Supporting Information

Supporting Information

Supporting Information

Supporting Information

Supporting Information

Supporting Information

Supporting Information

Supporting Information

Supporting Information

Supporting Information

Supporting Information

Supporting Information

Supporting Information
