# Supplementary material for: Loss of lysosomal acid lipase contributes to Alzheimer's disease pathology and cognitive decline
Source: Alzheimers Dement. 2025 Jul 18;21(7):e70486. doi: 10.1002/alz.70486 (PMC12271982; doi:10.1002/alz.70486)
Supplement: Supplementary file 2 — Supporting Information [file ALZ-21-e70486-s012.docx]

**Supplemental Table 1.** Demographics of postmortem human brain subjects. HC-Healthy controls, AUD-Alcohol Use Disorder, LOAD-Late onset Alzheimer’s disease

| **Human Brain Donors** | | | | | | | | |
| --- | --- | --- | --- | --- | --- | --- | --- | --- |
| **Cohort/ Comparison** | **Bank** | **Age** | **Sex** | **Dx** | **PMI (hrs)** | **Lifetime EtOH (kg)** | **Cause of Death** | |
| A | NSW | 24 | M | HC | 43 | 15 | Cardiac | |
| A | NSW | 37 | M | HC | 14.5 | 0 | Cardiac | |
| A | NSW | 37 | M | HC | 24 | 158 | Unknown | |
| A | NSW | 37 | M | HC | 28 | 1 | Cardiac | |
| A | NSW | 39 | M | HC | 22 | 0 | Unknown | |
| A | NSW | 40 | M | HC | 27 | 47 | Cardiac | |
| A | NSW | 40 | M | HC | 59 | 34 | Vascular | |
| A | NSW | 44 | M | HC | 50 | 28 | Cardiac | |
| A | NSW | 46 | M | HC | 29 | 115 | Cardiac | |
| A | NSW | 48 | M | HC | 24 | 17 | Cardiac | |
| A | NSW | 50 | M | HC | 30 | 0 | Cardiac | |
| A | NSW | 50 | M | HC | 40 | 18 | Cardiac | |
| A | NSW | 53 | M | HC | 16 | 102 | Cardiac | |
| A | NSW | 60 | M | HC | 28 | 0 | Cardiac | |
| A | NSW | 62 | M | HC | 46 | 5 | Cardiac | |
| A | NSW | 69 | M | HC | 52 | 273 | Cardiac | |
| A | NSW | 73 | M | HC | 9 | 245 | Cancer | |
| A | NSW | 75 | M | HC | 34 | 453 | Cardiac | |
| A | NSW | 76 | M | HC | 18 | 0 | Renal | |
| A | NSW | 80 | M | HC | 12 | 664 | Respiratory | |
| A | NSW | 25 | M | AUD | 43.5 | 552 | Toxicity | |
| A | NSW | 40 | M | AUD | 40 | 239 | Toxicity | |
| A | NSW | 40 | M | AUD | 50.5 | 5210 | Cardiac | |
| A | NSW | 41 | M | AUD | 48 | 409 | Cardiac | |
| A | NSW | 41 | M | AUD | 38.5 | 327 | Toxicity | |
| A | NSW | 41 | M | AUD | 32 | 1931 | Hepatic | |
| A | NSW | 42 | M | AUD | 41 | 1472 | Toxicity | |
| A | NSW | 44 | M | AUD | 15 | 639 | Cardiac | |
| A | NSW | 45 | M | AUD | 18.5 | 1799 | Respiratory | |
| A | NSW | 49 | M | AUD | 44 | 1012 | Cardiac | |
| A | NSW | 49 | M | AUD | 16 | 613 | Cardiac | |
| A | NSW | 50 | M | AUD | 17 | 2453 | Cardiac | |
| A | NSW | 50 | M | AUD | 34.5 | 5212 | Respiratory | |
| A | NSW | 61 | M | AUD | 59 | 8052 | Cardiac | |
| A | NSW | 61 | M | AUD | 23.5 | 5621 | Cardiac | |
| A | NSW | 71 | M | AUD | 9.5 | 6329 | Cardiac | |
| A | NSW | 71 | M | AUD | 39 | 1470 | Cardiac | |
| A | NSW | 73 | M | AUD | 43.5 | 782 | Cardiac | |
| A | NSW | 75 | M | AUD | 50.5 | 1789 | Cardiac | |
| A | NSW | 81 | M | AUD | 36 | 1635 | Infection | |
| B | NSW | 32 | F | AUD | 62 | 36135 | Toxicity | |
| B | NSW | 40 | M | AUD | 50.5 | 6406 | Cardiac | |
| B | NSW | 50 | M | AUD | 34.5 | 3660 | Respiratory | |
| B | NSW | 51 | F | AUD | 37 | 4987 | Toxicity | |
| B | NSW | 61 | M | AUD | 59 | 5811 | Cardiac | |
| B | NSW | 73 | F | AUD | 60 | 3331 | Cardiac | |
| B | NSW | 75 | M | AUD | 50.5 | 1622 | Cardiac | |
| B | NSW | 82 | F | AUD | 28 | 5559 | Hepatic | |
| **Cohort/ Comparison** | **Bank** | **Age** | **Sex** | **Dx** | **PMI** | **EtOH (kg)** | **Braak Stage** | **Cause of Death** |
| B, C | NSW | 44 | M | HC | 50 | 69 | 0 | Cardiac |
| B, C | NSW | 45 | F | HC | 29.5 | 15 | 0 | Cardiac |
| B, C | NSW | 67 | F | HC | 15.5 | 0 | 0 | Cancer |
| B, C | NSW | 69 | F | **HC** | 39 | 782 | III | Cardiac |
| B, C | NSW | 73 | M | HC | 38.5 | 48 | II | Cardiac |
| B, C | NSW | 76 | M | HC | 18 |  | I | Renal |
| B, C | NSW | 83 | M | HC | 10 | 64 | II | Respiratory |
| B, C | NSW | 85 | F | HC | 10 | 559 | II | Respiratory |
| **Cohort/ Comparison** | **Bank** | **Age** | **Sex** | **Dx** | **PMI** | **Braak Stage or**  **Disease Duration (yrs)** | **Cause of Death** | |
| C | NSW | 29 | F | HC | 40 | 0 | Cardiac | |
| C | NSW | 37 | M | HC | 14.5 | 0 | Cardiac | |
| C | NSW | 50 | M | HC | 34 | II | Cardiac | |
| C | NSW | 51 | F | HC | 41 | I | Toxicity | |
| C | NSW | 60 | M | HC | 28 | 0 | Cardiac | |
| C | NSW | 60 | F | HC | 36 | 0 | Cardiac | |
| C | NSW | 63 | F | HC | 42 | 0 | Cardiac | |
| C | NSW | 65 | M | HC | 50 | I | Cardiac | |
| C | NSW | 68 | M | HC | 31 | I | Cardiac | |
| C | NSW | 69 | M | HC | 16 | I | Cardiac | |
| C | NSW | 69 | M | HC | 52 | 0 | Cardiac | |
| C | NSW | 70 | F | HC | 6 | I-II | Renal | |
| C | NSW | 73 | M | HC | 9 | I | Cancer | |
| C | NSW | 75 | F | HC | 68 | I | Neuro | |
| C | NSW | 80 | M | HC | 12 | 0 | Respiratory | |
| C | NSW | 81 | M | HC | 29 | II | Cardiac | |
| C | NSW | 81 | F | HC | 35 | I | Cancer | |
| C | NSW | 88 | M | HC | 9 | II | Respiratory | |
| C | NSW | 88 | F | HC | 30 | II | Respiratory | |
| C | NSW | 91 | F | HC | 12 | I-II | Respiratory | |
| C | NSW | 74 | M | LOAD | 19 | V-VI | Infection | |
| C | NSW | 74 | F | LOAD | 16.5 | IV | Cardiac | |
| C | NSW | 75 | M | LOAD w/ vascular disease | 28 | V-VI | Neuro | |
| C | NSW | 78 | F | LOAD | 6 | V-VI | Cardiac | |
| C | NSW | 80 | M | LOAD | 8 | III | Respiratory | |
| C | NSW | 85 | F | LOAD w/ vascular disease | 16 | IV | Cardiac | |
| C | NSW | 89 | M | LOAD | 23.5 | III | Cardiac | |
| C | NSW | 90 | F | LOAD | 19 | V-VI | Cardiac | |
| C | NSW | 92 | F | LOAD | 24 | V-VI | Cardiac | |
| C | NSW | 92 | F | LOAD w/ vascular disease | 45.5 | VI | Neuro | |
| C | NSW | 93 | M | LOAD | 50 | V-VI | Cardiac | |
| C | VBB | 54 | M | LOAD | 49.5 | 5 | Respiratory | |
| C | VBB | 57 | F | LOAD | 6.5 | 9 | Respiratory | |
| C | VBB | 60 | F | LOAD | 60.5 | 7 | Neuro | |
| C | VBB | 61 | M | LOAD | 39.5 | 11 | Respiratory | |
| C | VBB | 63 | F | LOAD | 36 | 9 | Respiratory | |
| C | VBB | 64 | M | LOAD | 43 | 4 | Respiratory | |
| C | VBB | 64 | M | LOAD | 40.5 | 18 | Respiratory | |
| C | VBB | 65 | F | LOAD | 21 | 12 | Respiratory | |
| C | VBB | 67 | F | LOAD | 40.5 | 79 | Neuro | |
| C | VBB | 67 | M | LOAD | 39 | 13 | Respiratory | |
| C | VBB | 67 | F | LOAD | 72.5 | 15 | Respiratory | |
| C | VBB | 68 | M | LOAD | 47.5 | 15 | u/k | |
| C | VBB | 71 | F | LOAD | 38.5 | 13 | Neuro | |
| C | VBB | 72 | F | LOAD | 25 | 7 | Neuro | |
| C | VBB | 72 | F | LOAD | 41 | 5 | Respiratory | |
| C | VBB | 73 | M | LOAD | 19 | 13 | Sepsis | |
| C | VBB | 73 | M | LOAD | 9.5 | 10 | Respiratory | |
| C | VBB | 75 | M | LOAD | 40 | 9 | Respiratory | |
| C | VBB | 75 | M | LOAD | 83 | 16 | Respiratory | |
| C | VBB | 76 | F | LOAD | 15 | 14 | Dehydration | |
| C | VBB | 77 | M | LOAD | 58.5 | 10 | Cardiac | |
