# Supplementary material for: Loss of lysosomal acid lipase contributes to Alzheimer's disease pathology and cognitive decline
Source: Alzheimers Dement. 2025 Jul 18;21(7):e70486. doi: 10.1002/alz.70486 (PMC12271982; doi:10.1002/alz.70486)
Supplement: Supplementary file 3 — Supporting Information [file ALZ-21-e70486-s009.docx]

**Supplemental Table 2.** Key material resources and primer sequences

| **REAGENT or RESOURCE** | | | | |
| --- | --- | --- | --- | --- |
| **Antibodies** | **Isotype** | **Dilution** | **Clone** | **SOURCE; IDENTIFIER** |
| Akt (pan) | Rabbit IgG | 1:1000 (WB) | Mono | Cell Signaling; 4685S |
| APP (human, 6E10) | Mouse IgG | 1:500 (IF) | Mono | Biolegend; 803004 |
| APP [mouse, Y188] | Rabbit IgG | 1:1000 (WB) | Mono | Abcam; ab32136 |
| Aβ_1-42_ | Rabbit IgG | 1:500 (IHC) | Mono | Invitrogen; 700254 |
| Aβ_1-42_ | Rabbit IgG | 1:1000 (WB) | Mono | Abcam; ab201060 |
| BACE1 | Rabbit IgG | 1:1000 (WB) | Mono | Abcam; ab183612 |
| Beclin-1 | Rabbit IgG | 1:1000 (WB) | Mono | Cell Signaling; 3738S |
| CDK5 [EP715Y] | Rabbit IgG | 1:2000 (WB) | Mono | Abcam; ab40773 |
| FOXO1 | Rabbit IgG | 1:50 (CHIP) | Mono | Cell Signaling; 2880 |
| GAPDH | Mouse IgG | 1:2000 (WB) | Mono | Cell Signaling; 97166S |
| GAPDH | Chicken IgG | 1:5000 (WB) | Poly | Millipore; AB2302 |
| GAPDH | Mouse IgG | 1:2000 (WB) | Mono | Cell Signaling; 97166S) |
| GAPDH (14C10) | Rabbit IgG | 1:10,000 (WB) | Mono | Cell Signaling; 2118S |
| GSK3 alpha/beta (p-Tyr279) | Rabbit IgG | 1:1000 (WB) | Poly | Invitrogen; 36646 |
| GSK3β | Rabbit IgG | 1:1000 (WB) | Mono | Cell Signaling; 12456S |
| GSK3β (p-Ser9) | Rabbit IgG | 1:1000 (WB) | Mono | Cell Signaling; 5558S |
| Iba1 | Rabbit IgG | 1:1000 (IHC) | Poly | Wako; 019-1974 |
| LAL | Rabbit IgG | 1:250 (IF), (WB) | Poly | Novus; NBP1-54155 |
| LAMP1 | Rabbit IgG | 1:100 (IF) | Mono | Abcam; ab208943 |
| LAMP1 | Rabbit IgG | 1:1000 (IHC, WB) | Mono | Abcam; ab208943 |
| LC3A/B | Rabbit IgG | 1:1000 (WB) | Mono | Cell Signaling; 12741S |
| MAP2 | Chicken IgG | 1:500 (IF) | Poly | Abcam; ab5392 |
| Normal Rabbit IgG | Rabbit IgG | 1:50 (CHIP) | - | Cell Signaling; 2729S |
| Phospho-Akt (Thr308) | Rabbit IgG | 1:1000 (WB) | Mono | Cell Signaling; 2965S |
| PKA C-α | Rabbit IgG | 1:1000 (WB) | Mono | Cell Signaling; 4782S |
| PP2a [E155] | Rabbit IgG | 1:5000 (WB) | Mono | Abcam; ab32104 |
| PSEN-1 [EP000Y] | Rabbit IgG | 1:1000 (WB) | Mono | Abcam; ab76083 |
| p-tau (Ser202, Thr205) AT8 | Rabbit IgG | 1:500 (WB) | Poly | Invitrogen; MN1020 |
| p-tau (Ser214) | Rabbit IgG | 1:500 (WB) | Poly | Invitrogen; 44-742G. |
| phospho-Rpb1 (Pol II) | Rabbit IgG | 1:50 (CHIP) | Mono | Cell Signaling; 13499 |
| Tau [TAU-5] | Mouse IgG | 1:1000 (WB) | Mono | Abcam; ab80579 |
| Tau[pThr181] | Rabbit IgG | 1:200 (IHC), 1:500 (WB) | Poly | Novus; NB1008-2245 |
| TRAIL | Rabbit IgG | 1:500 (IHC) | Poly | Abcam; ab42121 |
| **Primers** | **Forward 5’🡪 3’** | | **Reverse 5’ 🡪 3’** | |
| 18S | GGTAACCCGTTGAACCCCAT | | CAACGCAAGCTTATGACCCG | |
| ABCA1 | AAAACCGCAGACATCCTTCAG | | CATACCGAAACTCGTTCACCC | |
| ABCA2 | CCCGTCATGCAGTCGCTTT | | CACTGGGTCGAACAAATTGCC | |
| ABCA5 | GATGTGGGAGTTTGGAGACAG | | GCTGAATTTGTCCATAGGGCT | |
| ABCG1 | CTTTCCTACTCTGTACCCGAGG | | CGGGGCATTCCATTGATAAGG | |
| Acox1 | TAACTTCCTCACTCGAAGCCA | | AGTTCCATGACCCATCTCTGTC | |
| ATF4 | ATGGCGCTCTTCACGAAATC | | ACTGGTCGAAGGGGTCATCAA | |
| ATP6v0c | ACTTATCGCTAACTCCCTGACT | | ACACCAGCATCTCCGACGA | |
| ATP6v0b | AGTTGCTCTACCTCGGGATCT | | ATGCCACATCAAAGCGAAAGC | |
| ATP6v0d1 | GCTACTTGGAGGGATTAGTGCG | | GCGGAACTCTACTACCATCTTCT | |
| ATP6v1a | CTACCCAAAATCCGCGATGAG | | CCATGTCACCTTCCAATCGAA | |
| ATP6v1b2 | ATGCGGGGAATCGTGAACG | | AGGCTGGGATAGGTAGTTCCG | |
| ATP6v1d | GGCAAAGACCGGATTGAAATCT | | GTCGAAATCGAAGAGTTAAGGCA | |
| ATP6ap2 | CTGGTGGCGGGTGCTTTAG | | GCTACGTCTGGGATTCGATCT | |
| ATP6v1h | GGATGCTGCTGTCCCAACTAA | | TCTCTTGCTTGTCCTCGGAAC | |
| APOE | CTCCCAAGTCACACAAGAACTG | | CCAGCTCCTTTTTGTAAGCCTTT | |
| APP (human) | GTCCAGAATGGGAAGTGGGA | | CACTGCATGTCTCTTTGGCG | |
| B2M | TTCTGGTGCTTGTCTCACTGA | | CAGTATGTTCGGCTTCCCATTC | |
| C3 | CAGGACGTGAGAGTCGATGG | | CTCTGCCTATGCTGCCTTCA | |
| CGI-58 | TGGTGTCCCACATCTACATCA | | CAGCGTCCATATTCTGTTTCCA | |
| CHOP | CTGGAAGCCTGGTATGAGGAT | | CAGGGTCAAGAGTAGTGAAGGT | |
| FOXO1 (human) | GGATGTGCATTCTATGGTGTACC | | TTTCGGGATTGCTTATCTCAGAC | |
| Gpnmb | GCTGGTCTTCGGATGAAAATGA | | CCACAAAGGTGATATTGGAACCC | |
| GRN | GGACACATGGCCTAGAATAACG | | AGACACACCCTTAGAGAACGG | |
| GRP78 | ACTTGGGGACCACCTATTCCT | | ATCGCCAATCAGACGCTCC | |
| HSL | GATTTACGCACGATGACACAGT | | ACCTGCAAAGACATTAGACAGC | |
| IFNα | TACTCAGCAGACCTTGAACCT | | CAGTCTTGGCAGCAAGTTGAC | |
| Igals | GGAGAGGGAATGATGTTGCCT | | TCCTGCTTCGTGTTACACACA | |
| IL-1β | CTGGTGTGTGACGTTCCCATTA | | CCGACAGCACGAGGCTTT | |
| IL-6 | GGCCTTCCCTACTTCACAAG | | ATTTCCACGATTTCCCAGAG | |
| Lcad | TCTTTTCCTCGGAGCATGACA | | GACCTCTCTACTCACTTCTCCAG | |
| *LIPA* (human) | TCTGGACCCTGCATTCTGAG | | CACTAGGGAATCCCCAGTAAGAG | |
| *LIPA* (mouse) | TGTTCGTTTTCACCATTGGGA | | CGCATGATTATCTCGGTCACA | |
| LPL | GGGAGTTTGGCTCCAGAGTTT | | TGTGTCTTCAGGGGTCCTTAG | |
| LXRα | CTGATTCTGCAACGGAGTTGT | | GACGAAGCTCTGTCGGCTC | |
| LXRβ | ATGTCTTCCCCCACAAGTTCT | | GACCACGATGTAGGCAGAGC | |
| MAGL | AGGCGAACTCCACAGAATGTT | | ACAAAAGAGGTACTGTCCGTCT | |
| MAPT (human) | CCAAGTGTGGCTCATTAGGCA | | CCAATCTTCGGACTGGACTCTGT | |
| Mcad | AGGGTTTAGTTTTGAGTTGACGG | | CCCCGCTTTTGTCATATTCCG | |
| MCP-1 | CCAGCCTACTCATTGGGA | | GGGCCTGCTGTTCACAGTT | |
| PERK | GCGTCGGAGACAGTGTTTG | | CGTCCATCTAAAGTGCTGATGAT | |
| PLIN | CTGTGTGCAATGCCTATGAGA | | CTGGAGGGTATTGAAGAGCCG | |
| PLIN2 | GACCTTGTGTCCTCCGCTTAT | | CAACCGCAATTTGTGGCTC | |
| PLIN3 | ATGTCTAGCAATGGTACAGATGC | | CGTGGAACTGATAAGAGGCAGG | |
| PLIN4 | GTGTCCACCAACTCACAGATG | | GGACCATTCCTTTTGCAGCAT | |
| PLIN5 | TGTCCAGTGCTTACAACTCGG | | CAGGGCACAGGTAGTCACAC | |
| PNPLA2 | TCCGTGGCTGTCTACTAAAGA | | TGGGATATGATGACGTTCTCTCC | |
| PPARα | AGAGCCCCATCTGTCCTCTC | | ACTGGTAGTCTGCAAAACCAAA | |
| PPARγ | GGAAGACCACTCGCATTCCTT | | GTAATCAGCAACCATTGGGTCA | |
| P2RY12 | CCCTGTGCGTCAGAGACTAC | | CAAGCTGTTCGTGATGAGCC | |
| Snap29 | TTCGACGATGACGTGGAAGAG | | GGTACTGCTGCCTGTCAATGG | |
| Srebp1 | TGACCCGGCTATTCCGTGA | | CTGGGCTGAGCAATACAGTTC | |
| Srebp2 | GCAGCAACGGGACCATTCT | | CCCCATGACTAAGTCCTTCAACT | |
| Stx17 | AAGTATCAGCGGTGCAGAATTT | | CCTTGACAGGATCTATCATTCGC | |
| TGFβ | CTCCCGTGGCTTCTAGTGC | | GCCTTAGTTTGGACAGGATCTG | |
| TLR4 | GCCTTTCAGGGAATTAAGCTCC | | AGATCAACCGATGGACGTGTAA | |
| Tmem119 | TCTTCCGGCAGTACGTGATG | | CGGCGCAGACTATGAACATGA | |
| TNFα | GACCCTCACACTCAGATCATCTTCT | | CCTCCACTTGGTGGTTTGCT | |
| TRAIL | GGGAGTCCTCTCGGAAAGG | | CCGGATAGCTGGTGTACTTGTA | |
| TREM2 | CTCCAGGAATCAAGAGACCTCC | | CCGGGTCCAGTGAGGATCT | |
| TRPML1 | CTGACCCCCAATCCTGGGTAT | | GGCCCGGAACTTGTCACAT | |
| VAMP8 | GGGAGTGCCGGAAATGACC | | TGAAGTGTTCAGACGTGGCTT | |
| YKT6 | AGTCAACTGATTGTGGAACGC | | TCTGGAAGGGTATTCGCTGTC | |
| **Primer sequences for CHIP analysis** | | | | |
| *LIPA* Pre-promoter  (89255992-89256016) | TCTGACAGAGACTGTTGCTCAACTT | | AGCTGGGGCATTATTGGATGAA | |
| *LIPA* Promoter  (89252180-89252199) | AAGCGCTTTAAATGGAGCCC | | CTTCTGCGCCTGACAGAGAG | |
| *LIPA* Exon 3  (89246089-89246108) | TATTTGTGCCCGGTAGGCAG | | TTCACGCTTGTGTTCCACCT | |
| *LIPA* Exon 6  (89224681-89224700) | AGTGTTAGGGCACACGGAAG | | CCCACGCAAACAATACCACC | |
| *LIPA* Exon 7  (89224681-89224700) | TGAGGGTGACCTAACAACGC | | CCTCCCTGAACAGAGGTTCG | |
| *LIPA* Exon 8 | TCAATGCCACCTTAATGCTGTT | | CCTGGAATGCCTACCTGGC | |
| *LIPA* Exon 10 (89214060-89214079) | TCATGGCAGGCCATAGTTCT | | GCGGGCTCCAAGAGTACATT | |
| Human LIPA | AAGCGCTTTAAATGGAGCCC | | CTTCTGCGCCTGACAGAGAG | |
| **Bacterial and virus strains** | | | | |
| AAV9.CD68.hM4di | | VectorBuilder | | |
| PHP.eB-syn-scrambledRNA | | VectorBuilder | | |
| PHP.eB-syn-shCON (10^12^ vg/mouse) | | VectorBuilder | | |
| PHP.eB-syn-WPRE-LAL (10^12^ vg/mouse) | | VectorBuilder | | |
| PHP.eB-syn-shLAL[1]-shLAL[2]-shLAL[3] (10^12^ vg/mouse) | | VectorBuilder | | |
| **Compounds** | | | | |
| DA4-JC | | MedChemExpress | | HY-P3255 |
| Genistein | | Sigma | | G6649 |
| LAListat-2 | | Tocris | | 6099 |
| Lysotracker red | | Thermo Fisher | | L-7528 |
| Recombinant LAL/sebelipase | | Creative Biomart | | THP-0136 |
| Protein A Dynabeads | | Thermo Fisher | | 10008D |
| **Experimental models: Organisms/strains** | | | | |
| Mouse: C57BL6/J WT | | Jackson Laboratory | | Stock No: 000664 |
| Mouse: 3xTg-AD | | MMRRC | | Stock No: 034830 (JAX: 004807) |
| Mouse: 5xFAD | | MMRRC | | Stock No: 034840 (JAX: 006554) |
| Mouse: CX3CR1.Cre^ERT2^ | | Jackson Laboratory | | Stock No: 021160 |
| Standard chow | | LabDiet PicoLab® | | Select 50 IF/6F, 5V5R |
| Western Diet | | Envigo | | TD.88137 |
