# Supplementary material for: Loss of lysosomal acid lipase contributes to Alzheimer's disease pathology and cognitive decline
Source: Alzheimers Dement. 2025 Jul 18;21(7):e70486. doi: 10.1002/alz.70486 (PMC12271982; doi:10.1002/alz.70486)
Supplement: Supplementary file 4 — Supporting Information [file ALZ-21-e70486-s004.docx]

| **Supplemental Table 3.** 2-way ANOVAs sex x Treatment for Supplemental Figures not otherwise listed in the legends. | | | | |
| --- | --- | --- | --- | --- |
| **RT PCR Measurements** | **Treatment Effect (DFn, DFd)** | **p value** | **Sex Effect F (DFn, DFd)** | **p value** |
| LAL (Supp. Fig. 2C) | F (2, 38) = 10.37 | P=0.0003 | F (1, 38) = 0.1403 | P=0.7101 |
| Cst7 (Supp. Fig. 2C) | F (2, 36) = 11.07 | P=0.0002 | F (1, 36) = 0.04508 | P=0.8330 |
| PLIN (Supp. Fig. 3N) | F (2, 30) = 4.486 | P=0.0198 | F (1, 30) = 0.1884 | P=0.6674 |
| PLIN2 (Supp. Fig. 3N) | F (2, 30) = 11.68 | P=0.0002 | F (1, 30) = 0.4345 | P=0.5148 |
| PLIN3 (Supp. Fig. 3N) | F (2, 23) = 4.262 | P=0.0266 | F (1, 23) = 0.04400 | P=0.8357 |
| PLIN 4 (Supp. Fig. 3N) | F (2, 32) = 17.04 | P<0.0001 | F (1, 32) = 6.746 | P=0.0141 |
| PLIN 5 (Supp. Fig. 3N) | F (2, 31) = 8.696 | P=0.0010 | F (1, 31) = 2.102 | P=0.1572 |
| CGI-58 (Supp. Fig. 3N) | F (2, 31) = 17.33 | P<0.0001 | F (1, 31) = 2.032 | P=0.1640 |
| ABCG1 (Supp. Fig. 3N) | F (2, 32) = 21.47 | P<0.0001 | F (1, 32) = 2.082 | P=0.1588 |
| ABCG4 (Supp. Fig. 3N) | F (2, 35) = 6.177 | P=0.0050 | F (1, 35) = 0.03773 | P=0.8471 |
| ABCA1 (Supp. Fig. 3N) | F (2, 31) = 11.09 | P=0.0002 | F (1, 31) = 0.4608 | P=0.5023 |
| ABCA2 (Supp. Fig. 3N) | F (2, 32) = 0.1899 | P=0.8279 | F (1, 32) = 0.005774 | P=0.9399 |
| ABCA5 (Supp. Fig. 3N) | F (2, 32) = 0.2338 | P=0.7928 | F (1, 32) = 1.292 | P=0.2642 |
| PERK (Supp. Fig. 3N) | F (1, 19) = 16.87 | P=0.0006 | F (1, 19) = 0.3888 | P=0.5403 |
| **IF/IHC Assessments** | **Treatment, Age, or Diagnosis Effect** | **p value** | **Sex Effect** | **p value** |
| Microglial Lipid (Supp. Fig. 3B) | F (1, 8) = 23.71 | P=0.0012 | F (1, 8) = 3.471 | P=0.0995 |
| %Microglial Lipid (Supp. Fig. 3C) | F (1, 8) = 11.89 | P=0.0087 | F (1, 8) = 13.54 | P=0.0062 |
| Lipid in WT ENT (Supp. Fig. 3G) | F (1, 14) = 11.88 | P=0.0039 | F (1, 14) = 1.902 | P=0.1895 |
| Lysosomal Lipid in WT ENT (Supp. Fig. 3H) | F (1, 14) = 13.49 | P=0.0025 | F (1, 14) = 0.07187 | P=0.7925 |
| Tau in LAMP1 (Supp. Fig. 3K) | F (1, 24) = 6.564 | P=0.0171 | F (1, 24) = 9.557 | P=0.0050 |
| Total Lipid in FCX with age (Supp. Fig. 4A) | F (2, 29) = 84.71 | P<0.0001 | F (1, 29) = 21.41 | P<0.0001 |
| Total Lipid in ENT with age (Supp. Fig. 4B) | F (2, 22) = 34.64 | P<0.0001 | F (1, 22) = 7.204 | P=0.0136 |
| Total LAL in ENT with age (Supp. Fig. 4D) | F (2, 30) = 17.11 | P<0.0001 | F (1, 30) = 3.599 | P=0.0675 |
| Total LAL in FCX with age (Supp. Fig. 4E) | F (2, 29) = 20.64 | P<0.0001 | F (1, 29) = 3.944 | P=0.0566 |
| Neuronal LAL in FCX with age (Supp. Fig. 4F) | F (2, 28) = 2.587 | P=0.0931 | F (1, 28) = 14.60 | P=0.0007 |
| Human Aβ1-42 in ENT (Supp. Fig. 8A) | F (1, 34) = 21.97 | P<0.0001 | F (1, 34) = 0.9483 | P=0.3370 |
| Human Aβ1-42 in CA (Supp. Fig. 8C) | F (1, 48) = 21.56 | P<0.0001 | F (1, 48) = 0.5578 | P=0.4588 |
| Human Aβ1-42 in SUB (Supp. Fig. 8E) | F (1, 33) = 13.14 | P=0.0010 | F (1, 33) = 4.662 | P=0.0382 |
| Human Aβ1-42 in Dentate (Supp. Fig. 8G) | F (1, 46) = 19.01 | P<0.0001 | F (1, 46) = 2.867 | P=0.0972 |
| **Western Blot Assessments** | **Treatment Effect F (DFn, DFd)** | **p value** | **Sex Effect F (DFn, DFd)** | **p value** |
| GSK3β (Supp. Fig. 1G) | Isoform x Tx F (2, 96) = 7.556 | P=0.0009 | Isoform x Sex F (2, 96) = 7.556 | P=0.0009 |
