# Supplementary material for: Loss of lysosomal acid lipase contributes to Alzheimer's disease pathology and cognitive decline
Source: Alzheimers Dement. 2025 Jul 18;21(7):e70486. doi: 10.1002/alz.70486 (PMC12271982; doi:10.1002/alz.70486)
Supplement: Supplementary file 5 — Supporting Information [file ALZ-21-e70486-s006.docx]

| **Supplemental Table 4. Pearson’s Coefficients for Lysosomal Lipid in 3xTg-AD.**  **p<*0.05, ***p<*0.01, ****p<*0.001, *****p<*0.0001 Dunnett’s post-test 1-way ANOVA | | |
| --- | --- | --- |
| **Region** | **Sex** | **Pearson’s Coefficient**  **(mean ±SEM)** |
| Frontal Cortex  (3xTg-AD mice) | Male | Control: 0.26 ± 0.02  Obese: 0.38 ± 0.04*  EtOH: 0.31 ± 0.01 |
|  | Female | Control: 0.27 ± 0.02  Obese: 0.33 ± 0.04***  EtOH: 0.32 ± 0.01* |
| Entorhinal Cortex  (3xTg-AD mice) | Male | Control: 0.26 ± 0.01  Obese: 0.38 ± 0.02*  EtOH: 0.34 ± 0.02* |
|  | Female | Control: 0.22 ± 0.01  Obese: 0.38 ± 0.02****  EtOH: 0.33 ± 0.01**** |
| Frontal Cortex  (WT mice) | Male | 3 mo: 0.13 ± 0.03  12 mo: 0.29 ± 0.02*  20 mo: 0.41 ± 0.05*** |
|  | Female | 3 mo: 0.02 ± 0.01  12 mo: 0.25 ± 0.04***  20 mo: 0.59 ± 0.01**** |
| Entorhinal Cortex  (WT mice) | Male | 3 mo: 0.03 ± 0.005  12 mo: 0.42 ± 0.02*  20 mo: 0.44 ± 0.09*** |
|  | Female | 3 mo: 0.04 ± 0.006  12 mo: 0.30 ± 0.03****  20 mo: 0.56 ± 0.02**** |
