# Supplementary figures and images for: Loss of lysosomal acid lipase contributes to Alzheimer's disease pathology and cognitive decline
Source: Alzheimers Dement. 2025 Jul 18;21(7):e70486. doi: 10.1002/alz.70486 (PMC12271982; doi:10.1002/alz.70486)

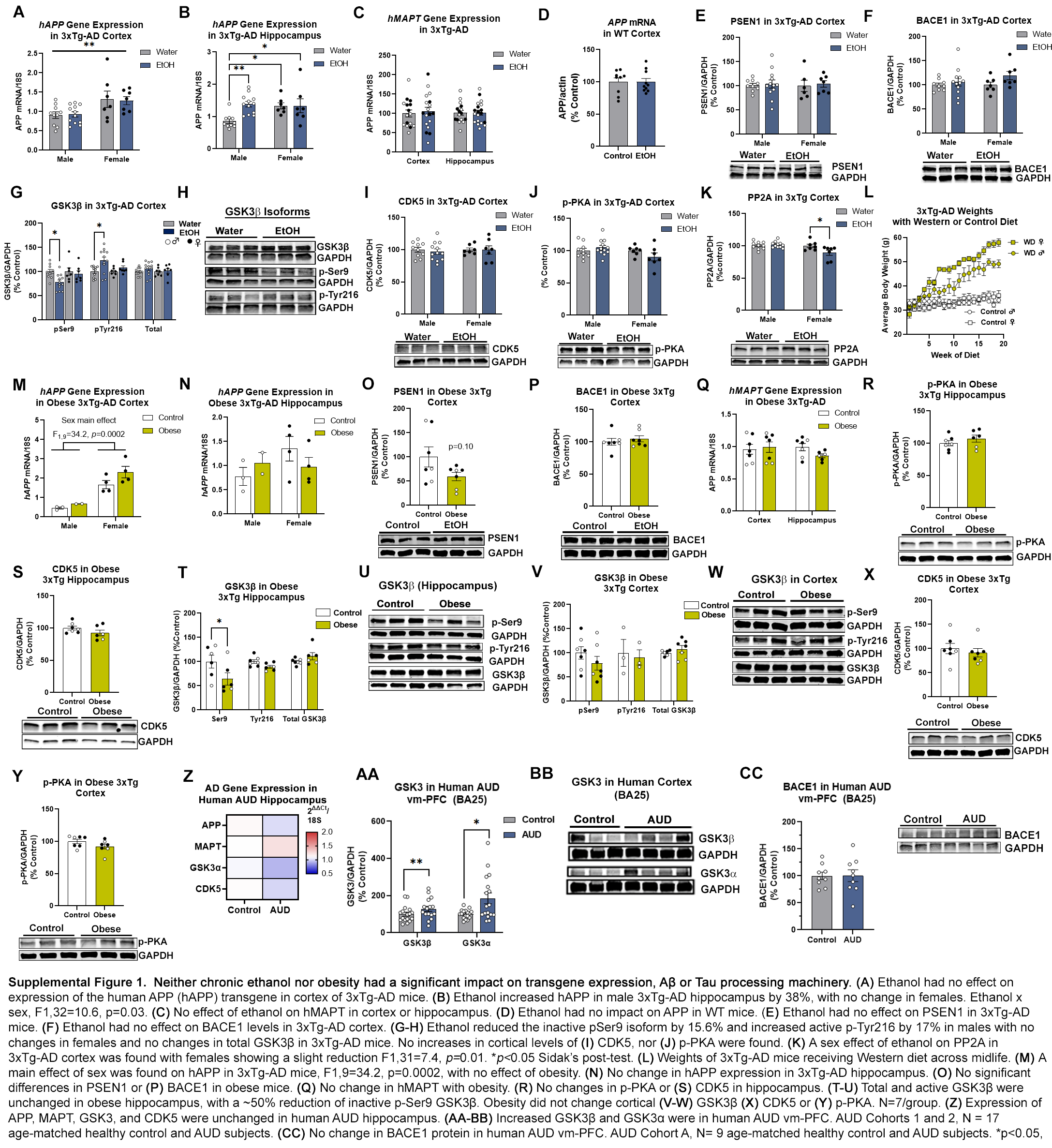

Supplement: Supplementary file 6 — Supporting Information [file ALZ-21-e70486-s014.tif]

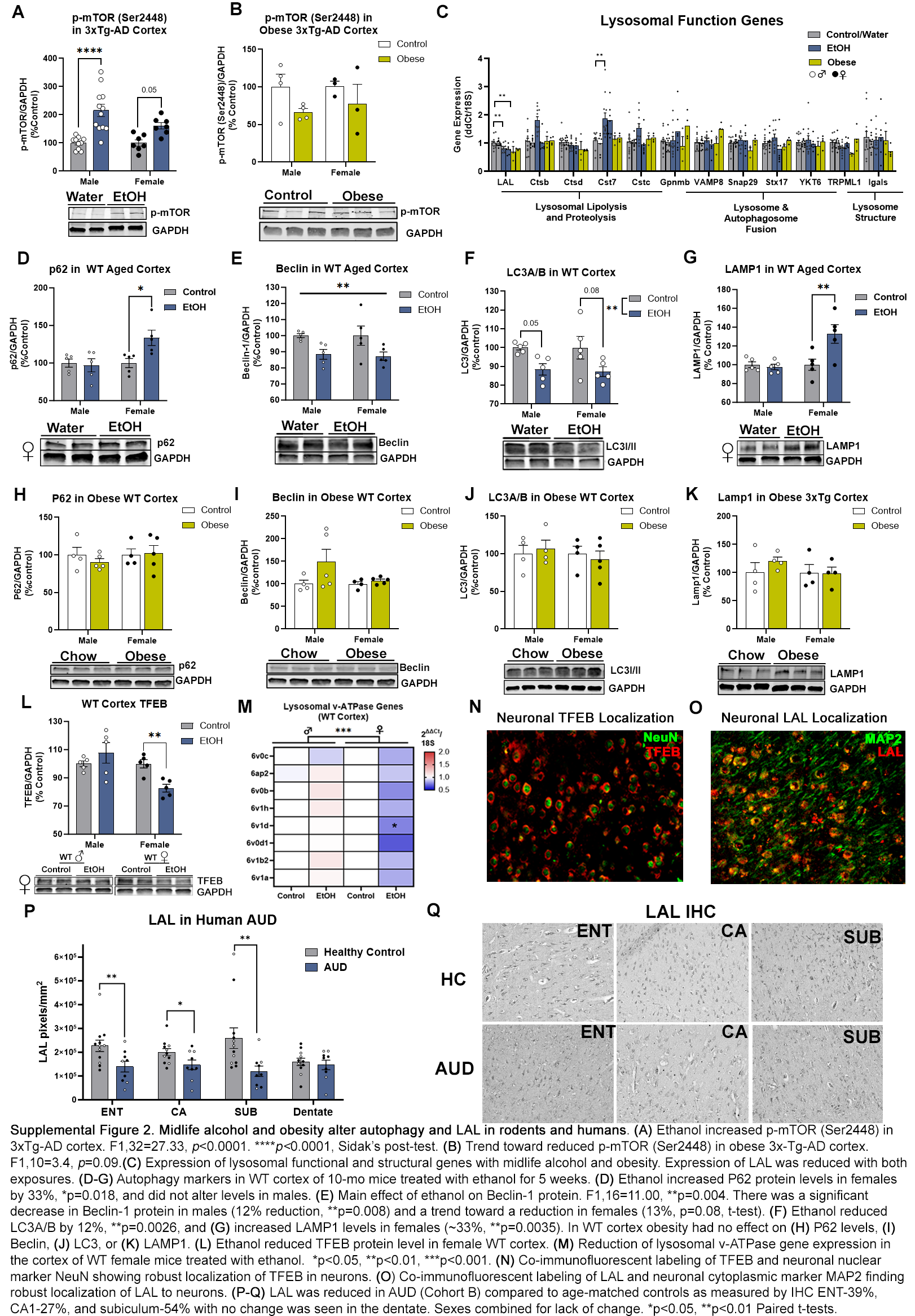

Supplement: Supplementary file 7 — Supporting Information [file ALZ-21-e70486-s003.tif]

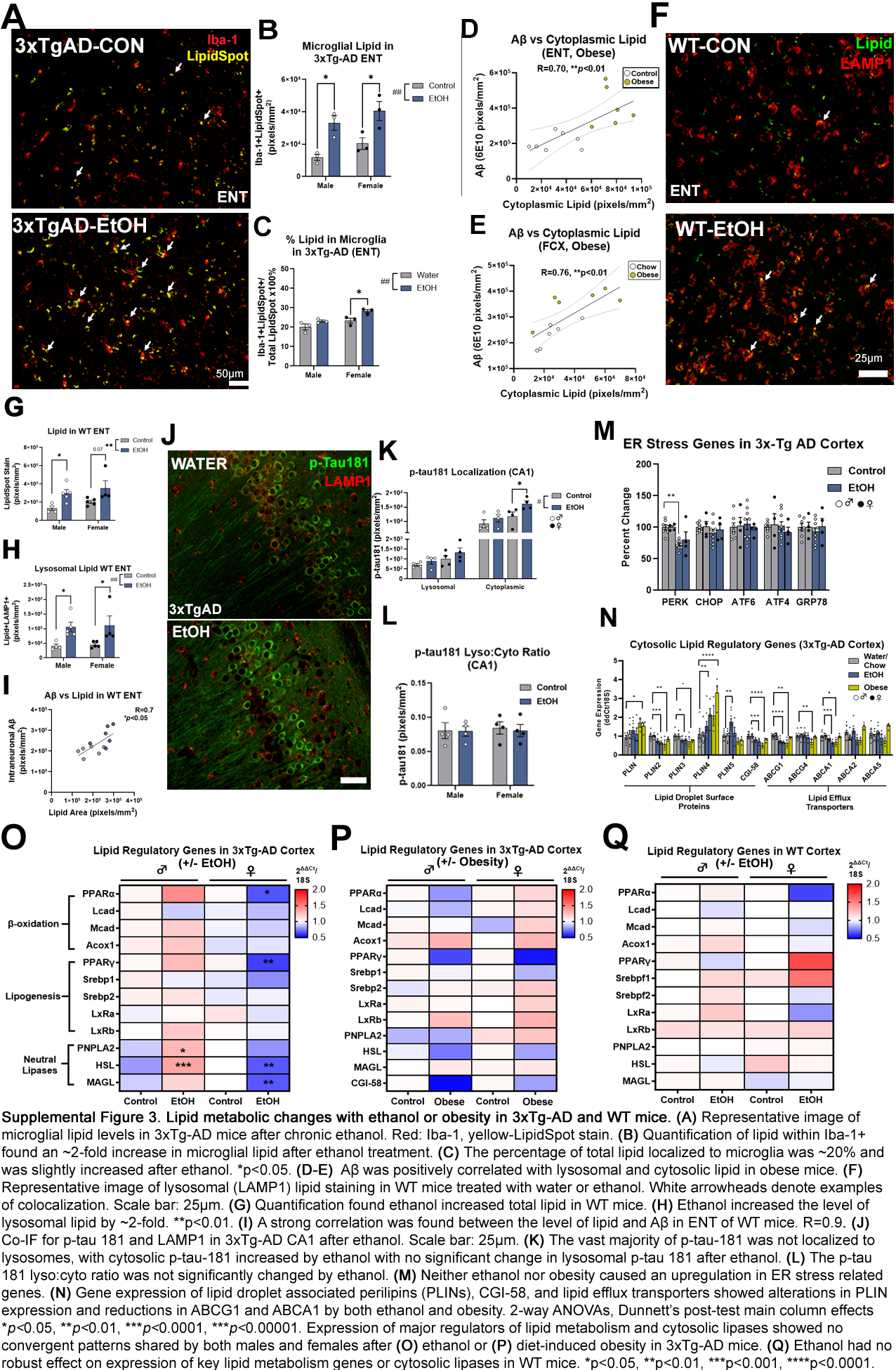

Supplement: Supplementary file 8 — Supporting Information [file ALZ-21-e70486-s002.tif]

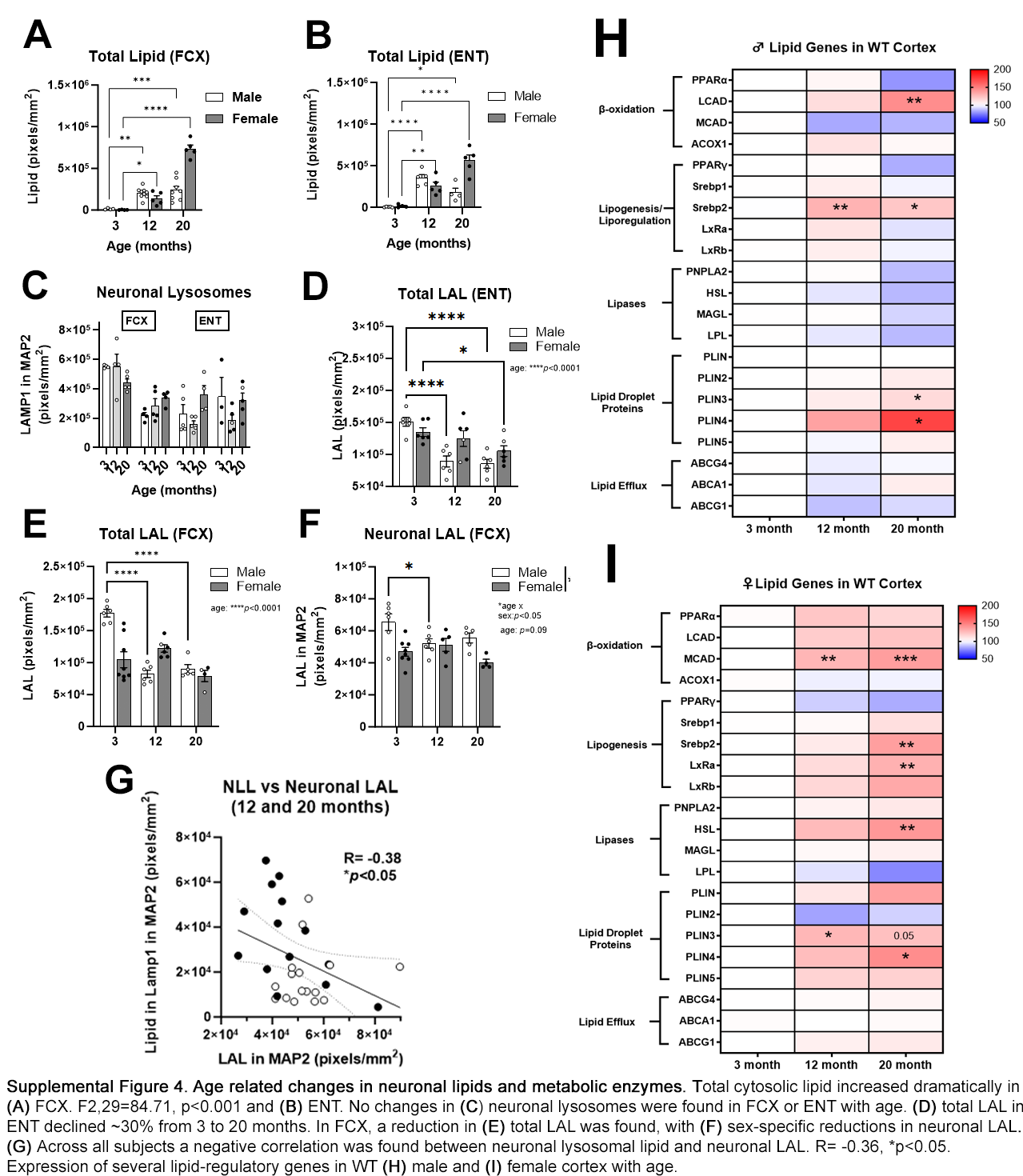

Supplement: Supplementary file 9 — Supporting Information [file ALZ-21-e70486-s010.tif]

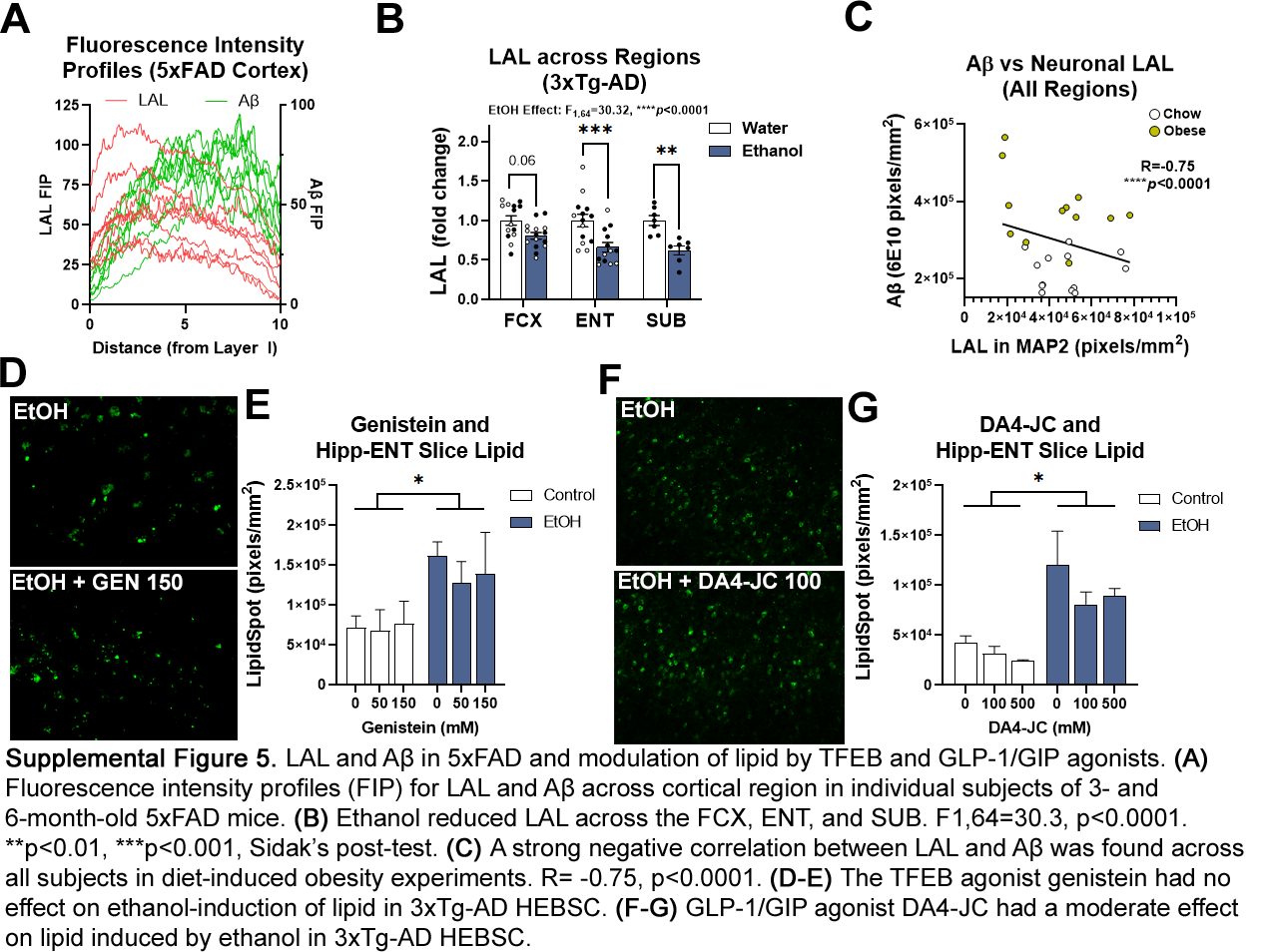

Supplement: Supplementary file 10 — Supporting Information [file ALZ-21-e70486-s011.tif]

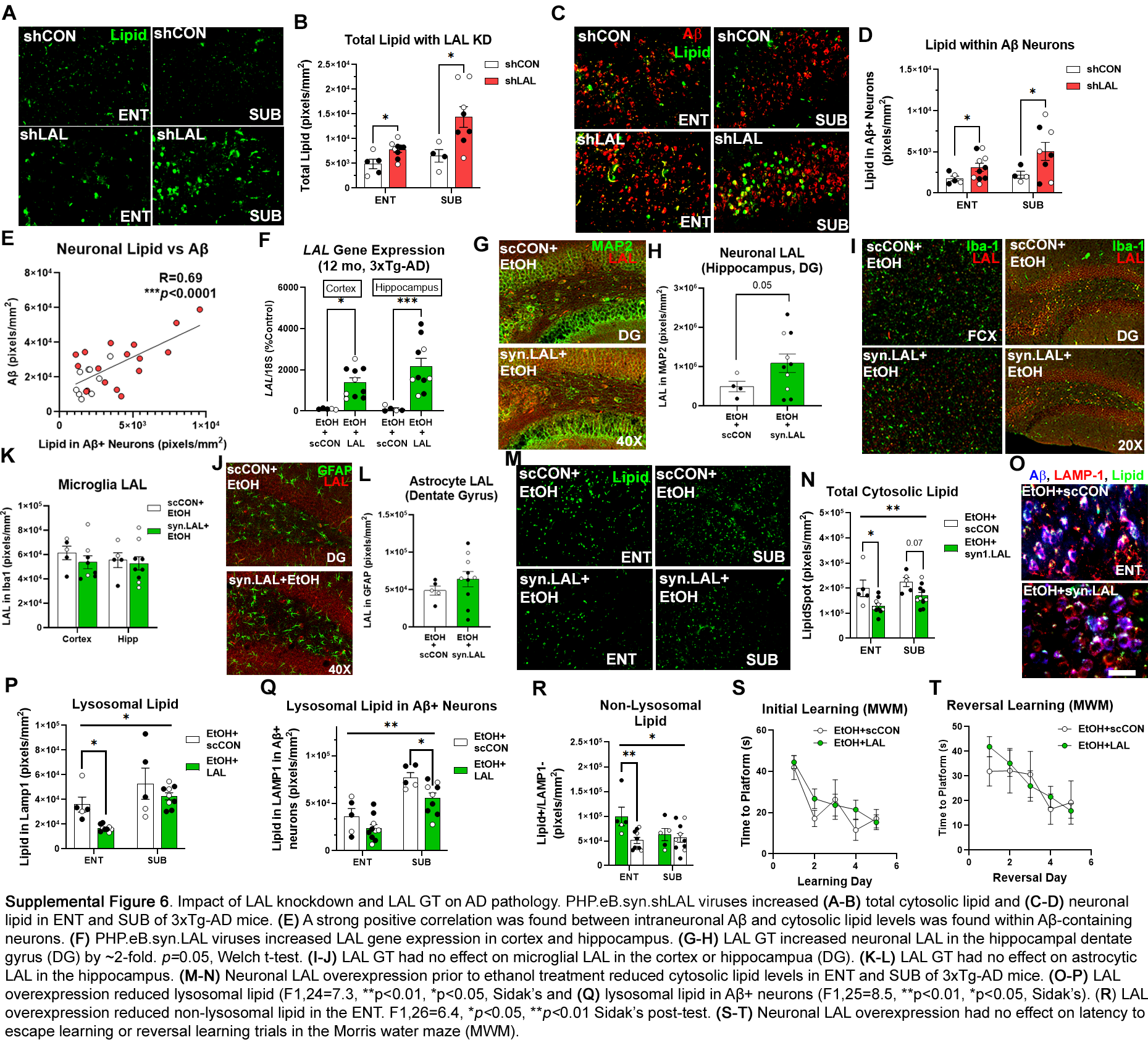

Supplement: Supplementary file 11 — Supporting Information [file ALZ-21-e70486-s013.tif]

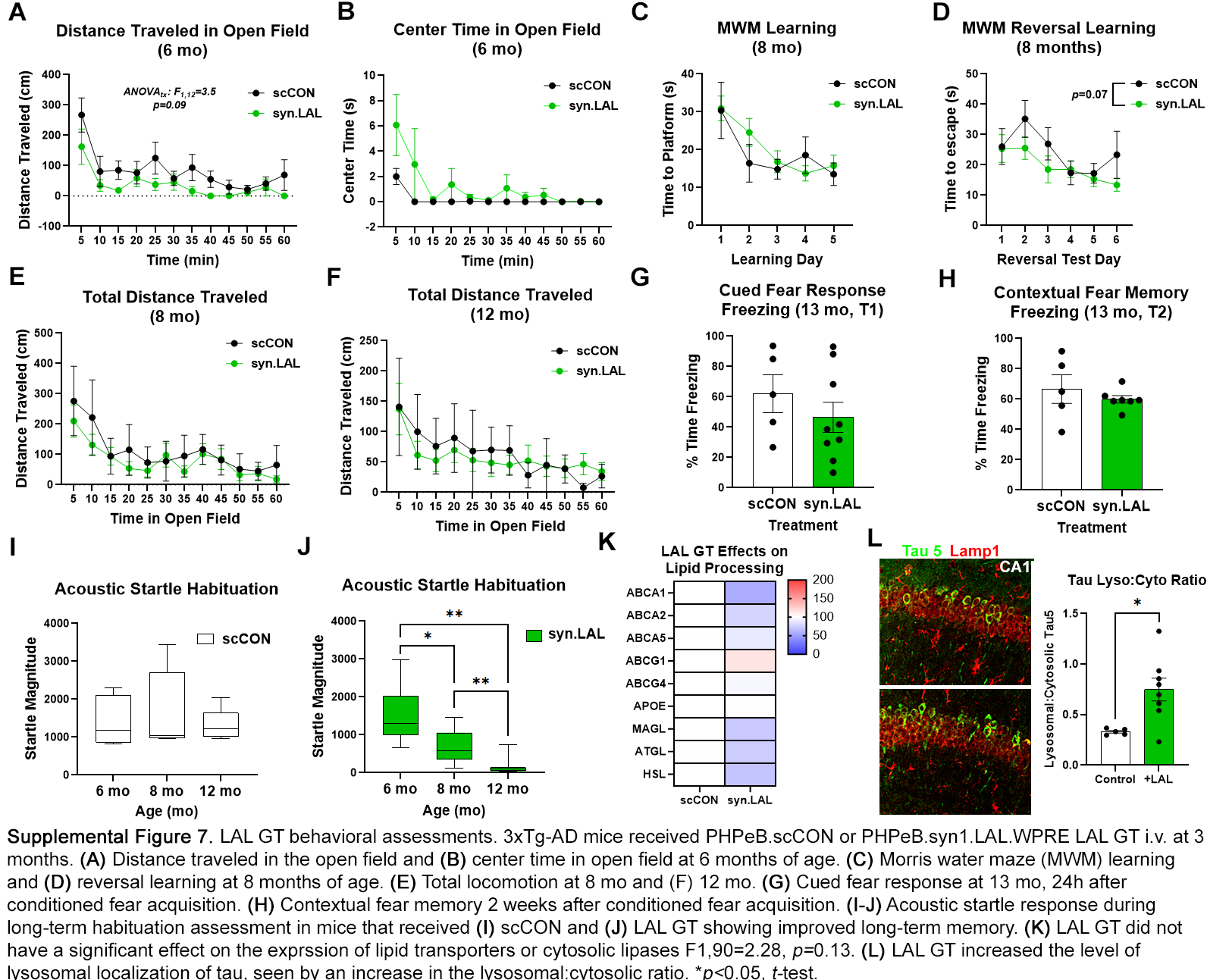

Supplement: Supplementary file 12 — Supporting Information [file ALZ-21-e70486-s007.tif]

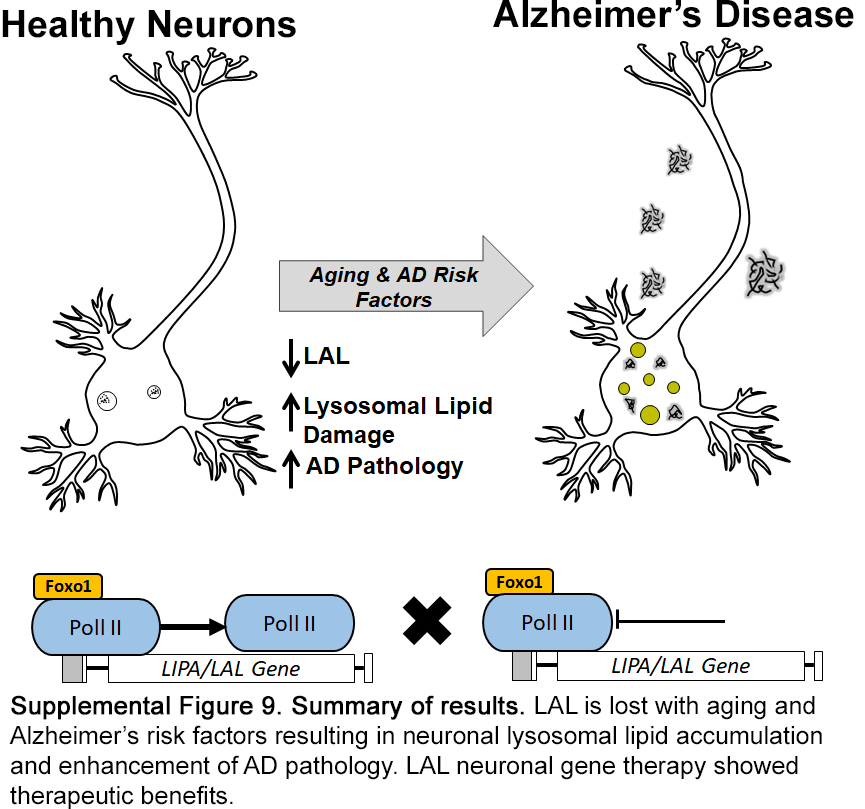

Supplement: Supplementary file 14 — Supporting Information [file ALZ-21-e70486-s001.tif]
